# TRBP ensures efficient Dicer processing of precursor microRNA in RNA-crowded environments

**DOI:** 10.1038/ncomms13694

**Published:** 2016-12-09

**Authors:** Mohamed Fareh, Kyu-Hyeon Yeom, Anna C. Haagsma, Sweeny Chauhan, Inha Heo, Chirlmin Joo

**Affiliations:** 1 Kavli Institute of NanoScience, Delft University of Technology, 2629HZ Delft, The Netherlands; 2 Department of BioNanoScience, Delft University of Technology, 2629HZ Delft, The Netherlands

## Abstract

The RNA-binding protein TRBP is a central component of the Dicer complex. Despite a decade of biochemical and structural studies, the essential functionality of TRBP in microRNA (miRNA) biogenesis remains unknown. Here we show that TRBP is an integral cofactor for time-efficient Dicer processing in RNA-crowded environments. We competed for Dicer processing of pre-miRNA with a large amount of cellular RNA species and found that Dicer-TRBP, but not Dicer alone, remains resilient. To apprehend the mechanism of this substrate selectivity, we use single-molecule fluorescence. The real-time observation reveals that TRBP acts as a gatekeeper, precluding Dicer from engaging with pre-miRNA-like substrates. TRBP acquires the selectivity using the PAZ domain of Dicer, whereas Dicer moderates the RNA-binding affinity of TRBP for fast turnover. This coordinated action between TRBP and Dicer accomplishes an efficient way of discarding pre-miRNA-like substrates.

## Abstract (web summary)

The RNA binding protein TRBP is a component of the Dicer complex but its role in microRNA biogenesis remains poorly understood. Here the authors use a crowded RNA environment and single-molecule imaging to show that TRBP acts as a gatekeeper to prevent Dicer engagement with pre miRNA-like substrates.

MicroRNA (miRNA) is small non-coding RNA that is ubiquitously expressed in higher eukaryotes. MiRNA biogenesis occurs through a series of enzymatic processing steps, including the cleavage of hairpin RNA (precursor miRNA or pre-miRNA) by Dicer proteins^1^. Human Dicer is a multi-domain enzyme that consists of several RNA-binding domains, including the PAZ domain and tandem RNase III domains^1^,^2^. The PAZ domain recognizes the 2-nucleotide (nt) 3′-overhang of the pre-miRNA, and the region between the PAZ and RNase III domains acts as a molecular ruler that defines the miRNA size^3^,^4^,^5^,^6^. In addition to having its own RNA-binding domains, Dicer associates with RNA-binding partners that assist in pre-miRNA processing and miRNA loading^7^,^8^,^9^,^10^. TRBP (transactivation response element RNA-binding protein) is an RNA-binding cofactor of Dicer complexes in human cells^7^,^8^. By being tightly associated with Dicer^11^, TRBP increases the RNA-binding affinity of Dicer^12^ and enhances cleavage accuracy^11^,^13^. However, recent TRBP knockout studies have suggested that TRBP is dispensable for miRNA biogenesis^11^,^13^,^14^ and have raised controversy over the cellular function of TRBP in miRNA biogenesis.

Here we demonstrate that TRBP is a critical factor that assists Dicer to effectively find and cleave pre-miRNA among a large amount of cellular RNAs. We used biochemical and single-molecule fluorescence techniques to reveal how TRBP coordinates pre-miRNA recognition, helping Dicer to discriminate pre-miRNA-like species. Our study suggests that TRBP recruits double-stranded RNA substrates regardless of the end structure of the RNA. TRBP positions the 3′-end of RNA to the PAZ domain of Dicer to verify the authenticity of the substrate. Non-canonical substrates lacking the 2-nt 3′-overhang are quickly released by TRBP, whereas canonical pre-miRNA is transferred to Dicer for cleavage. This selective loading by Dicer-TRBP promotes the efficient RNA processing in the RNA-crowded environment.

## Results

### TRBP ensures efficient Dicer processing

**Figure 1:**
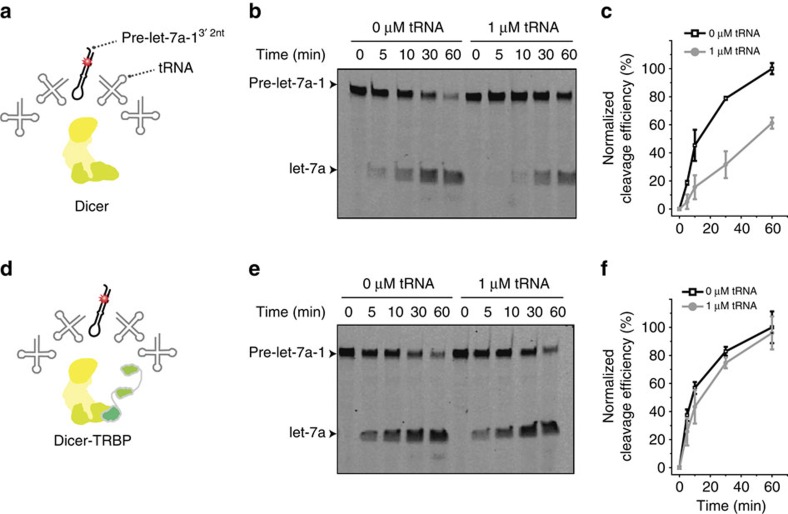
TRBP ensures efficient processing of pre-miRNA in an RNA-crowded cellular environment. (**a**,**d**) Schematic representation of *in vitro* cleavage in an RNA-crowded environment. (**b**) Time-course analysis of pre-let-7a-1^3′2nt^ cleavage by Dicer alone in absence and presence of 1 μM competitor tRNA. (**c**) Quantification of the cleavage efficiency of Dicer alone in absence (black) and presence of 1 μM competitor tRNA (grey). The efficiency was normalized to the highest efficiency observed. (**e**) Time-course analysis of pre-let-7a-1^3′2nt^ cleavage by Dicer-TRBP in absence and presence of 1 μM tRNA. (**f**) Quantification of the cleavage efficiency of Dicer-TRBP in absence (black) and presence of 1 μM competitor tRNA (grey). Error is the s.d. of three independent measurements.

Previous observations that recombinant Dicer proteins alone could process pre-miRNA substrates left the biological role of TRBP in miRNA biogenesis unclear^15^. We hypothesized that TRBP, the first reported in mammals but ill-characterized cofactor of Dicer, might have a prominent role when Dicer-TRBP encounters an RNA crowded cellular environment. To test this hypothesis, we mimicked the crowded environment by competing for Dicer processing of nanomolar pre-miRNA substrates (pre-let-7a-1^3′2nt^) with micromolar competitor RNAs (tRNA; Fig. 1). When Dicer was present alone, the cleavage was substantially inhibited by the competitor tRNA (Fig. 1a–c). However, TRBP-bound Dicer remained resilient to the excessive amount of tRNA (Fig. 1d–f). We confirmed this phenomenon by testing six different RNA strands, which showed a different degree of inhibition depending on their end structure (Supplementary Fig. 1a–f). A control using unlabelled canonical pre-let-7a-1^3′2nt^ as a competitor led to the expected reduction of the cleavage efficiency of both Dicer alone and Dicer-TRBP (Supplementary Fig. 1a–c). This led us to speculate that TRBP acts as a gatekeeper, precluding Dicer from engaging with cellular RNAs other than pre-miRNA.

**Figure 2:**
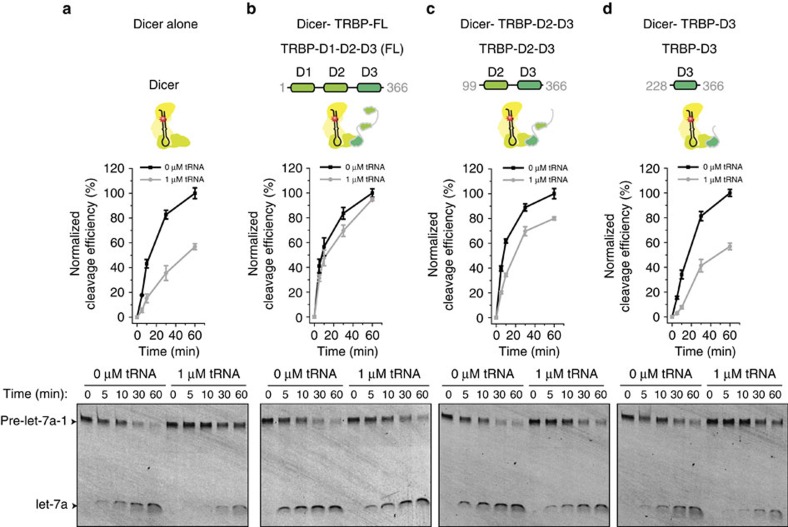
TRBP's dsRBDs mediate the processing of pre-miRNA in an RNA-crowded environment. (**a**–**d**) Time-course cleavage of pre-let-7a-1^3′2nt^ (1 nM) by Dicer alone (**a**), Dicer-TRBP-FL (**b**), Dicer-TRBP-D2-D3 (**c**) and Dicer-TRBP-D3 (**d**). The cleavage experiments were performed in absence (black) and presence (grey) of excess of tRNA (1 μM). The graphs at the bottom quantify the cleavage efficiency. The efficiency was normalized to the highest efficiency observed. Error is the s.d. of three independent experiments.

To understand this new function of TRBP, we generated TRBP mutants. TRBP consists of three different double-stranded RNA-binding domains (dsRBDs). The first two domains (domains 1 and 2) mediate an interaction with dsRNA molecules, while domain 3 anchors TRBP to Dicer and other cofactors^11^,^16^,^17^,^18^. We constructed two truncation mutants (D2–D3 and D3) containing domains 2–3 and domain 3, respectively (Fig. 2a–d). Western blotting and immunofluorescence analysis confirmed homogenous cellular expression and co-localization of Dicer and TRBP (Supplementary Fig. 2a–d). Whereas Dicer complexed with full-length TRBP (FL-TRBP) was insensitive to the excessive amount of the RNA competitor (Fig. 2b), Dicer alone and Dicer complexed with D3-TRBP exhibited a decrease by ∼50% in the cleavage efficiency (Fig. 2a,d). Dicer complexed with D2–D3 TRBP partially restored the cleavage efficiency (∼20% inhibition; Fig. 2c). Our biochemical data indicate the importance of the RNA-binding activity of TRBP in efficient processing of pre-miRNA in an RNA-crowded environment.

### TRBP makes an entry port for RNA in Dicer-TRBP

**Figure 3:**
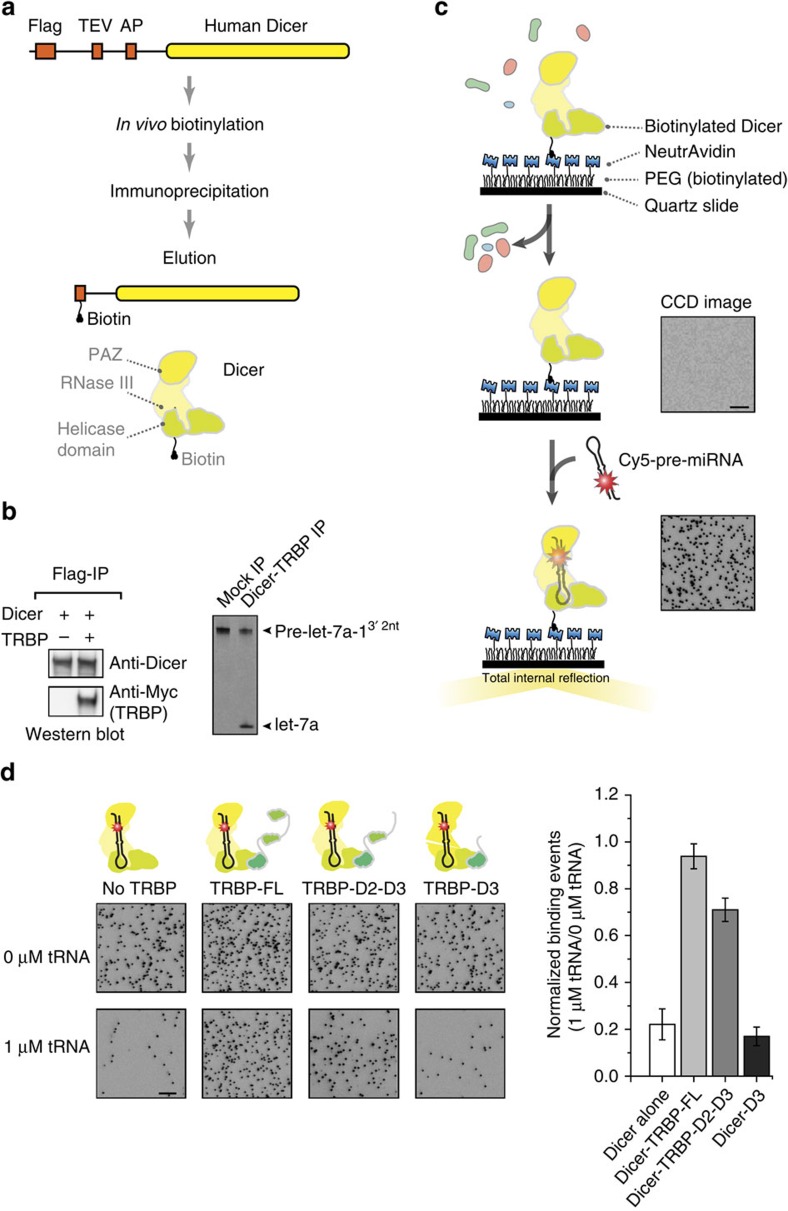
TRBP's dsRBDs mediate the recruitment of pre-miRNA in an RNA-crowded environment. (**a**) Schematic of sample preparation. Dicer was constructed with Flag, TEV and AP tags, which were used for immunoprecipitation, elution and biotinylation, respectively. Dicer proteins were biotinylated in HEK 293 cells. The proteins were immunoprecipitated using Flag-antibody beads. The proteins were eluted out of the beads via TEV cleavage. (**b**) Western blotting of Dicer IPs. Dicer proteins were expressed without and with Myc-TRBP and were pulled down using Flag beads (left). *In vitro* cleavage of pre-let-7a-1^3′2nt^ (right). Mock is a negative control with Flag-mCherry IPs. (**c**) Schematic of single-molecule immobilization. Dicer IPs were conjugated to a polymer-coated surface via NeutrAvidin–biotin interaction. Contaminant proteins were washed away before 200 pM Cy5-labelled pre-let-7a-1^3′2nt^ was introduced. Interactions between the surface-immobilized Dicer complexes with Cy5-labelled pre-miRNA were visualized through total internal reflection fluorescence (TIRF) microscopy. Dots in the CCD (charge-coupled device) image reflect docking of pre-let-7a-1^3′2nt^ to individual Dicer complexes. The CCD image illustrated the binding events over 25 × 25 μm^2^ field of view. Scale bar, 5 μm. (**d**) The CCD images in the left illustrate the stable docking of Cy-5-labelled pre-let-7a-1^3′2nt^ to surface-immobilized Dicer complexes in absence (upper panels) and presence of 1 μM competitor tRNA (bottom panels). The histogram in the right quantifies the inhibition of the pre-miRNA-binding activity due to the presence of 1 μM competitor tRNA. Error is the s.d. obtained from 10 different fields of view in three independent experiments. Scale bar, 5 μm.

We sought to observe how Dicer-TRBP interacts with pre-miRNA. Such a dynamic process is challenging to investigate using standard biochemical techniques and calls for real-time observation methods. We used single-molecule fluorescence techniques that can capture weak interactions with high temporal resolution. This required the purification of the Dicer-TRBP as a protein complex. We used SIMPlex (single-molecule approach to immunoprecipitated protein complexes), a technique that we previously developed^19^, but improved the purification and immobilization scheme by using biotin as a tag^20^. *In vivo* biotinylated Dicer complexes were pulled down using Flag-beads, and Dicer immunoprecipitates (IPs) were immobilized on a surface via biotin–NeutrAvidin conjugation (Fig. 3a–c). *In vivo* biotinylation of Dicer and biotin–NeutrAvidin conjugation did not affect Dicer activity (Supplementary Fig. 3a,b). When we needed to exclude RNA cleavage in single-molecule observations, we used a catalytically inactive Dicer (TN Dicer)^21^ while including magnesium ions. After RNA was introduced, several hundred spots per field of view appeared, indicating docking of Cy5-labelled pre-let-7a-1^3′2nt^ (our standard construct, Supplementary Fig. 3e) and other Cy5-labelled pre-miRNA substrates (Supplementary Fig. 3d). A control experiment without Dicer-TRBP showed no binding events (Supplementary Fig. 3d), thus excluding non-specific interactions between RNA and the passivated surface.

Using SIMPlex, we immobilized Dicer IPs on the surface with or without ectopic expression of TRBP and then quantified the number of docked RNA substrates (pre-let-7a-1^3′2nt^) per field of view. When an equivalent amount of IPs were used, Dicer lacking TRBP showed one order of magnitude fewer stably bound pre-let-7a-1^3′2nt^ (58±13 binding) compared with Dicer associated with TRBP (781±94 binding; Supplementary Fig. 3f). Similar results were obtained with other human pre-miRNA variants such as pre-miR-17 (Supplementary Fig. 3g). These data are consistent with previous reports that TRBP increases the pre-miRNA-binding affinity of Dicer^12^,^20^.

We used our single-molecule-binding assay to quantitatively investigate whether TRBP contributes to the binding of pre-miRNA to Dicer in an RNA-crowded environment, and whether the degree of binding was correlated with Dicer-processing efficiency shown in Figs 1 and 2. We introduced in the imaging chamber 200 pM of Cy5-labelled pre-let-7a-1^3′2nt^ with and without competitor tRNA (1 μM, three orders of magnitude higher concentration than pre-miRNA) and counted the number of encounters between surface-immobilized Dicer complexes and pre-let-7a-1^3′2nt^ (Fig. 3d). When tRNA was provided together with pre-let-7a-1^3′2nt^, the binding activity of Dicer-lacking TRBP was reduced by 77.9±6.5%. Dicer complex with FL-TRBP, however, was resilient to the competitor tRNA molecules and did not show any noticeable inhibition in the pre-miRNA-binding activity (6.1±5.3% inhibition). The truncation of TRBP's RNA-binding domain 1 (TRBP D2–D3) and both RNA-binding domains 1–2 (TRBP D3) reduced the pre-miRNA-binding affinity by 29.0±4.9% and 83.0±3.9%, respectively, when the competitor was added (Fig. 3d). Our real-time measurements further support the different degree of RNA-binding among Dicer-TRBP complexes (Supplementary Fig. 4). Our biochemical and single-molecule data indicate that TRBP, especially dsRBD 1 and 2, mediate efficient substrate recognition by Dicer in an RNA-crowded environment.

### TRBP modulates the energy landscape of Dicer–RNA interaction

**Figure 4:**
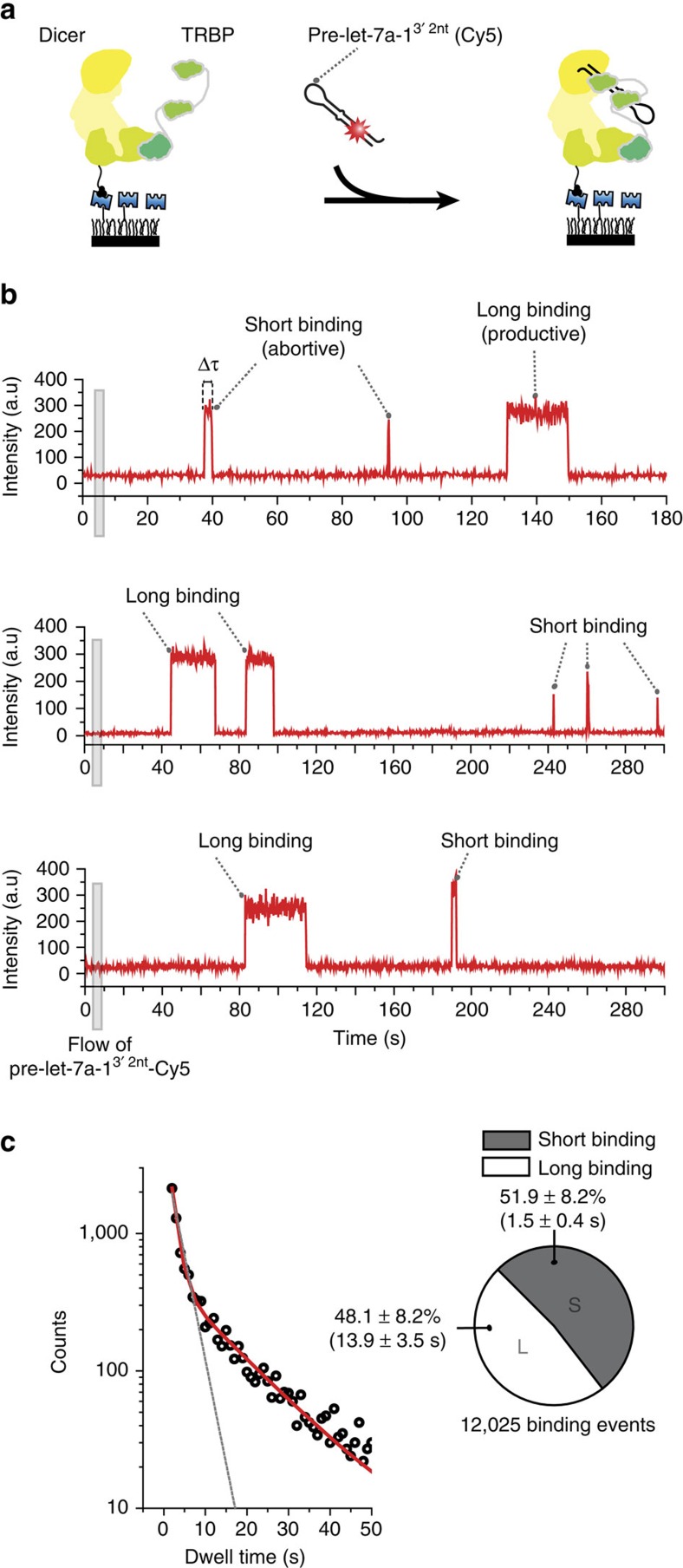
Real-time observation of pre-miRNA recognition by the Dicer-TRBP complex. (**a**) Schematic representation of a single-molecule assay to capture pre-miRNA recognition by the Dicer-TRBP complex in real time. (**b**) Representative time traces (a time resolution 300 ms) exhibiting recognition of multiple Cy5-labelled pre-let-7a-1^3′2nt^ by a single Dicer-TRBP complex. The dwell time (Δ*τ*) is the time between docking and dissociation. Cy5-labelled pre-let-7a-1^3′2nt^ was added at time 5 s. (**c**) Dwell-time histogram derived from binding events recorded for 450 s in a pre-steady-state condition. The distribution was fitted with a double-exponential decay (red line). The dashed grey line is a fit to a single-exponential decay. The pie chart displays the ratio between short binding (Δ*τ*_short_=1.5±0.4 s, grey) and long binding (Δ*τ*_long_=13.9±3.5 s, white). Error is the s.d. of four independent measurements.

To find out how TRBP contributes to the pre-miRNA selection during the substrate recognition, we measured the binding and dissociation kinetics in a pre-steady-state condition. After the introduction of 200 pM Cy5-labelled pre-let-7a-1^3′2nt^ into the imaging chamber (Fig. 4a), the docking to Dicer-TRBP was evident by the sudden appearance of fluorescence, which was followed by the disappearance of the signal (dissociation of pre-let-7a-1^3′2nt^). In the time traces, we noticed distinctively different interactions characterized by two dwell times when both TN (Fig. 4b) and wild-type Dicer (Supplementary Fig. 5e,f) were used. To understand the heterogeneity, we built a dwell-time histogram using 12,025 docking events that were obtained from 8,459 time traces during the first 450 s of observation (Fig. 4c). The dwell-time distribution did not follow a single exponential decay (grey dashed line, *R*^2^=0.956) but instead followed a double-exponential decay (red line, *R*^2^=0.999). This result indicates that Dicer-TRBP exhibits two different binding modes for RNA recognition. A half of the interactions (51.9±8.2%) was short-lived (Δ*τ*_short_=1.5±0.4 s; Fig. 4c). The second population showed a more stable binding to the Dicer-TRBP complex (Δ*τ*_long_=13.9±3.5 s; Fig. 4c).

We measured the RNA-binding kinetics of Dicer alone and Dicer associated with different forms of TRBP (Supplementary Fig. 5a–d). Both short and long binding modes were observed in all cases. Together with the observation on the high RNA-binding affinity of Dicer-TRBP (Supplementary Fig. 3f,g), this indicates that Dicer protein alone defines the dual binding mode, but TRBP modulates the energy landscape of Dicer-RNA interactions for the efficient substrate recognition.

To assess the impact of RNA competitors on the two binding modes, we carried out pre-steady-state single-molecule experiments using an excessive amount of competitor tRNAs (1 μM). The total number of binding events was substantially reduced by the competitor RNAs when Dicer was not associated with FL-TRBP (Supplementary Fig. 6a–d,f), consistent with the result in Fig. 2. The competitor RNAs influenced the short and the long binding modes to the same degree (Supplementary Fig. 6e,f), implying that the two binding modes must be positioned on the same pathway of RNA recognition.

### Dicer-TRBP uses the PAZ domain for pre-miRNA recognition

**Figure 5:**
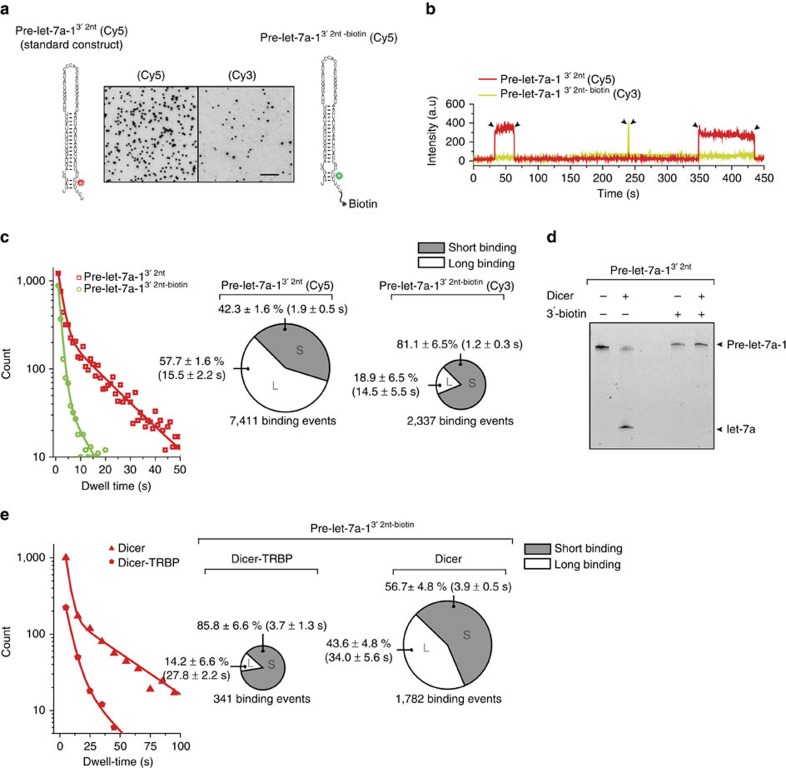
The role of the PAZ domain. (**a**) Two-colour competition assay. The standard pre-let-7a-1^3′2nt^ was labelled with Cy5. A biotin group was attached to the 3′-overhang of Cy3-labelled pre-let-7a-1 (pre-let-7a-1^3′2nt-biotin^). The charge-coupled device images show docking of standard pre-let-7a-1^3′2nt^ (left) and pre-let-7a-1^3′2nt-biotin^ (right). Scale bar, 5 μm. (**b**) Representative time trace (time resolution 300 ms) showing long binding of two standard pre-let-7a-1^3′2nt^ substrates (Cy5, red) and short binding of one pre-let-7a-1^3′2nt-biotin^ substrate (Cy3, green) to a single Dicer-TRBP complex. (**c**) Dwell-time histograms derived from binding of standard pre-let-7a-1^3′2nt^ (red) and pre-let-7a-1^3′2nt-biotin^ (green). The distributions were fitted with a double-exponential decay. The pie chart in the left displays the percentage of short binding (Δ*τ*_short_=1.9±0.5 s, grey) and long binding (Δ*τ*_long_=15.5±2.2 s, white) obtained with Cy5-labelled standard pre-let-7a-1^3′2nt^. The pie chart in the right displays the percentage of short binding (Δ*τ*_short_=1.2±0.3 s, grey) and long binding (Δ*τ*_long_=14.5±5.5 s, white) obtained with Cy3-labelled pre-let-7a-1^3′2nt-biotin^. The size of the pie charts is proportional to the total number of binding events. Error is the s.d. of four independent measurements. (**d**) *In vitro* cleavage of standard pre-let-7a-1^3′2nt^ (left) and pre-let-7a-1^3′2nt-biotin^ (right) by wild-type Dicer-TRBP. The top arrow indicates pre-let-7a-1^3′2nt^, and the bottom arrow indicates a cleaved product (mature let-7a). (**e**) Dwell-time histograms derived from binding of pre-let-7a-1^3′2nt-biotin^ to Dicer alone (triangle) and Dicer-TRBP complex (pentagon). The distributions were fitted with a double-exponential decay. The pie chart in the left displays the percentage of short binding (Δ*τ*_short_=3.7±1.3 s, green) and long binding (Δ*τ*_long_=27.8±2.2 s, white) obtained with the Dicer-TRBP complex. The pie chart in the right displays the percentage of short binding (Δ*τ*_short_=3.7±0.5 s, green) and long binding (Δ*τ*_long_=34.0±5.6 s, white) obtained with Dicer alone. The size of the pie charts is proportional to the total number of binding events. Error is the s.d. of four independent measurements.

To comprehend how Dicer exploits the two binding modes for substrate recognition and how TRBP enhances the recognition mechanism, we focused on the interaction between Dicer-TRBP and RNA in a pre-steady-state condition. The PAZ domain of Dicer is critical for selecting RNA with 2-nt 3′-end. We perturbed the interaction between the 3′-end of the pre-miRNA and the PAZ domain by attaching a biotin group to the 3′-end (Fig. 5a and Supplementary Fig. 7a). This modification inhibited Dicer cleavage (Fig. 5d). This construct (pre-let-7a-1^3′2nt-biotin^) was labelled with Cy3 (green) and was flowed into an imaging chamber. To prevent the biotinylated RNA from being immobilized, unbound NeutrAvidin was saturated with 1 mM free biotin (Supplementary Fig. 7b). The standard construct (pre-let-7a-1^3′2nt^) with Cy5 (red) was added together at the same concentration as a reference. The docking events of these two substrates to Dicer-TRBP were recorded simultaneously using two laser beams (532 and 633 nm). The docking of pre-let-7a-1^3′2nt-biotin^ was 3.1 times less frequently observed (2,337 binding events) compared with that of the standard construct (7,411 events; Fig. 5a–c and Supplementary Movie 1). Intriguingly, the percentage of the long binding obtained with pre-let-7a-1^3′2nt-biotin^ was reduced from 57.7±1.6 to 18.9±6.5% among the total binding events (Fig. 5c). A similar observation was made with Dicer that has an altered 3′-binding pocket^22^ (Supplementary Fig. 7c,d). These observations suggest that the PAZ domain arbitrates the initial RNA selection process by sensing the termini of the encountered RNA molecule. The decreased percentage of long binding events in Fig. 5c indicates that this interaction is also crucial for long binding which is likely to lead to cleavage.

The reduced number of binding events observed with pre-let-7a-1^3′2nt-biotin^ hints that Dicer-TRBP discriminates pre-miRNA from pre-miRNA-like substrates at the initial recognition step, far faster than our time resolution (300 ms). To find out how Dicer probes pre-miRNA-like substrates when it is not associated with TRBP, we determined the RNA-binding kinetics of Dicer alone using pre-let-7a-1^3′2nt-biotin^. Strikingly, compared with Dicer-TRBP, Dicer alone exhibited a five times larger number of binding on this pre-miRNA-like substrate (Fig. 5e). The binding was characterized with a large fraction (56.7±4.8%) of cleavage-incompetent stable binding. These non-optimal kinetic properties—frequent and long-lived non-productive interactions with pre-miRNA-like substrates—elucidate the origin of the slower turnover of Dicer alone compared with Dicer-TRBP shown in Figs 1, 2, 3.

### Dicer-TRBP promptly rejects non-canonical pre-miRNA

We additionally examined how Dicer-TRBP distinguishes other pre-miRNA-like molecules. We tested a pre-let-7a-1 construct with a long 3′-tail (pre-let-7a-1^3′ U49^; Supplementary Fig. 8a), which marks pre-miRNA for degradation^23^. We expected that this long tail would interfere with the 3′-end recognition by the PAZ domain. Our data showed that it indeed led to a reduction in overall binding events by a factor of 2.5. Notably, the percentage of the long binding was also reduced from 54.7±4.0 to 31.0±3.6% of the total binding (Supplementary Fig. 8a,b). To further characterize the recognition of pre-miRNA by the PAZ domain, we tested a pre-let-7a-1 construct with a 1-nt overhang (pre-let-7a-1^3′ 1nt^, 72 nt), which is 1 nt shorter than the overhang in our standard construct (pre-let-7a-1^3′2nt^, 73 nt; Supplementary Fig. 8c,d). This family of pre-miRNA with 1-nt 3′-overhang is named group II pre-miRNA and is known to be a poorer substrate of Dicer than group I with 2-nt 3′-overhang^24^. We observed that the total binding decreased by a factor of 2.9, whereas the long binding events persisted (55.6±5.7%; Supplementary Fig. 8c,d). The differentiation of subtle structural features (1 nt) highlights the importance of the mono-uridylation of group II pre-miRNA for efficient RNA processing^24^. The oligo-U tail of pre-miRNA is, conversely, known to act as a degradation marker. The reduced binding frequency that we observed with oligo-uridylated pre-miRNA explains the molecular origin behind a previous observation that Dicer cannot process oligo-uridylated pre-miRNA^21^.

### TRBP recruits dsRNA without selectivity

**Figure 6:**
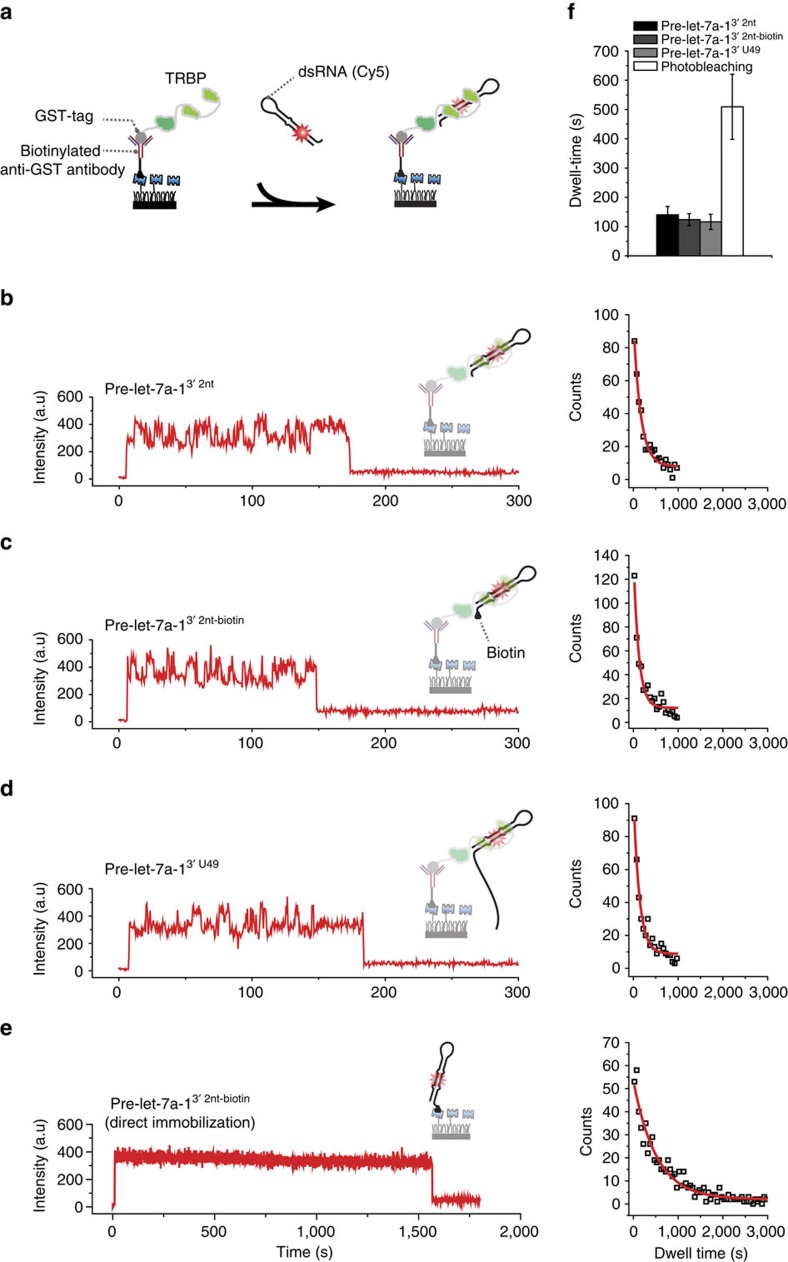
TRBP binds dsRNA without selectivity. (**a**) Schematic representation of a single-molecule assay to capture RNA recognition by TRBP in real time. TRBP was immobilized on a surface via a biotinylated anti-glutathione-*S*-transferase (GST) antibody. (**b**–**d**) Representative time traces (a time resolution 500 ms) that exhibit binding of Cy5-labelled RNA to a single TRBP protein. The histograms (right) indicate the distribution of the dwell time. The dwell time is the time between docking and dissociation. (**e**) A representative time trace from Cy5-labelled pre-let-7a-1^3′2nt-biotin^, which is immobilized on the surface via biotin–NeutrAvidin conjugation. The dwell time reflects the timescale of photobleaching. (**f**) Average dwell times from **b** to **e**. Error is the s.d. of four independent experiments.

**Figure 7:**
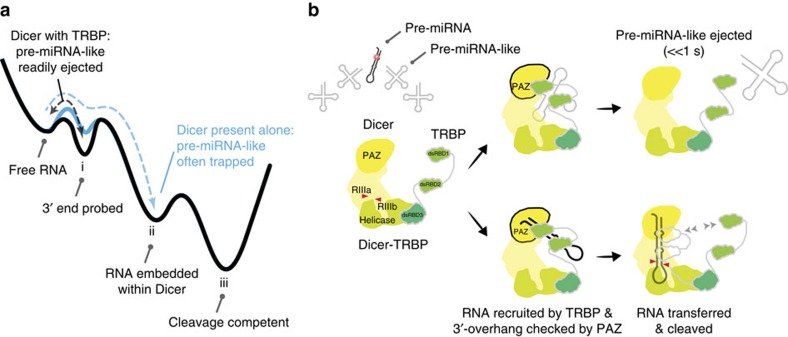
Model of pre-miRNA recognition by Dicer-TRBP. (**a**) Free energy landscape. ‘i' stands for a state in which the 3′-end of RNA is recognized. ‘ii' for a state in which RNA is embedded within Dicer. ‘iii' for the cleavage competent state of Dicer. (Blue) Energy landscape of Dicer-recognizing RNA. Cellular RNA such as tRNA easily falls into ‘ii', making Dicer processing inefficient. (Black) Energy landscape of Dicer-TRBP-recognizing RNA. The energy barrier between ‘free RNA' and ‘i' is lowered by TRBP. The energy level of ‘i' is deepened by TRBP. Cellular RNA such as tRNA is trapped in ‘i' and readily ejected out of Dicer-TRPB, making Dicer processing efficient in an RNA-crowded environment. (**b**) The dsRBDs of TRBP recruit pre-miRNA by interacting with the stem region of the RNA. TRBP relocates the dsRNA into Dicer, where the PAZ domain verifies the length of 3′-overhang. If the dsRNA molecule possesses pre-miRNA features, the RNA becomes stably associated and cleaved. If it does not, it is ejected far faster than 1 s. Dicer lacking TRBP partner fails in rejecting non-canonical pre-miRNA substrates rapidly, which compromises the enzyme turnover in an RNA-crowded environment.

We sought to assess whether TRBP additionally contributed to the observed substrate selectivity. We immobilized glutathione-*S*-transferase (GST)-tagged recombinant TRBP proteins on a quartz surface via an anti-glutathione-*S*-transferase (GST) antibody (Fig. 6a). We introduced 200 pM Cy5-labelled pre-let-7a-1^3′2nt^ into a microfluidic chamber and recorded the interactions between TRBP and pre-let-7a-1^3′2nt^ in real time (Fig. 6a). The recombinant TRBP proteins, which were not associated with Dicer, bound pre-let-7a-1^3′2nt^ for ∼120 s (Fig. 6b,f). This stable binding is in agreement with a previously published study^25^. The binding induced fluctuations in the fluorescence intensity—a characteristic of the dynamic movement of TRBP along dsRNA, which was reported earlier^26^,^27^. Using this single-molecule binding assay, we determined whether TRBP shows any difference in the binding affinity towards pre-miRNA-like substrates. When we tested pre-let-7a-1^3′2nt^ with a biotinylated 3′-end, we did not observe any noticeable difference in the dwell time (Fig. 6c,f). Furthermore, we did not observe any difference from oligo-uridylated pre-let-7a-1^3′ U49^ (Fig. 6d,f). We also performed photobleaching experiments using direct immobilization of the biotinylated pre-let-7a-1^3′2nt^, which confirmed that TRBP holds different dsRNA molecules for ∼120 s (Fig. 6e and Supplementary Fig. 9a–c). These results indicate that TRBP recruits pre-miRNA to Dicer by recognizing a double-stranded RNA structure but discerns the unique feature of pre-miRNA (2-nt 3′-overhang) by exclusively using the PAZ domain of Dicer. In Fig. 7, we propose how TRBP with high RNA-binding affinity but lacking substrate specificity can enhance the substrate recognition of Dicer-TRBP by modulating the energy landscape of Dicer–RNA interactions.

## Discussion

In the human cell, Dicer encounters a multitude of different RNA molecules—only 0.01% among 360,000 RNA molecules are miRNAs^28^. Despite a decade of biochemical and structural studies, the understanding of how Dicer recognizes its substrates fast but also accurately in the crowded cellular environment remains incomplete. We developed a single-molecule two-colour fluorescence technique, combined with single-molecule immunoprecipitation, and revealed the dynamic process of how the human Dicer-TRBP protein complex probes pre-miRNA. Our results suggest that Dicer-TRBP associates with and examines pre-miRNA in a defined order. This ordered recognition of pre-miRNA enables Dicer to efficiently cleave pre-miRNA even when the amount of cellular RNA molecules exceeds by three orders of magnitude.

For comprehensive understanding of our findings, we draw the energy landscape of Dicer–RNA interactions (Fig. 7a). The short binding mode (‘i' in the landscape) represents the entry of RNA to Dicer, which leads to a long binding mode (ii) and consequently to the cleavage competent state of the enzyme (iii). We determined the order of (i) and (ii) based on the data in Fig. 5 in which non-canonical pre-miRNA, not cleavable by Dicer, exhibited mainly the short binding mode. Dicer-TRBP can shortly interact with any dsRNA, yet only canonical pre-miRNA bypasses this entry check point (i) and reaches the long binding mode (ii). Additional support on our model is from Supplementary Fig. 6. When Dicer was challenged with three orders of magnitude excess of competitor RNAs, the long and short binding modes were equally influenced, indicating that these two binding modes are likely to be on the same reaction pathway.

The 3′-end of RNA is first recognized by the PAZ domain of Dicer in the short binding mode (i) within a few seconds. If the RNA has the canonical 3′ 2-nt overhang, the RNA is transferred to a more stable binding mode (ii). The RNA-binding affinity of Dicer was reduced when FL-TRBP was not associated (Supplementary Fig. 3f,g). We speculate that TRBP lowers the energy barrier between the free RNA state and the first binding state (i). The high RNA-binding affinity of TRBP also deepens the energy level of (i), which makes RNA difficult to go over the barrier between (i) and (ii). We hypothesize that these two alternations in the energy landscape makes RNA more readily associated with Dicer and also prevents cellular RNA from falling into (ii) as reflected by the rapid rejection (Fig. 5c). This energy landscape makes even canonical pre-miRNA rejected with ∼50% of a probability, but this landscape is obligatory to efficiently displace pre-miRNA-like RNA. It should be noted that not all molecules at (ii) are subject to cleavage (iii). As shown in Fig. 5e, a large amount of non-canonical pre-miRNA was held stably but not cleaved. There must be an additional barrier towards cleavage, which represent a final checkup of the pre-miRNA cleavage.

Our single-molecule data provide insights into how TRBP and Dicer coordinate each other for RNA recognition (Fig. 7b). TRBP, a double-stranded RNA-binding protein^29^, is conserved in many invertebrate and vertebrate species, suggesting an important function in the RNA interference pathway^29^. TRBP contains dsRBD1 and dsRBD2, which have α-β-β-β-α-fold—a common motif of a dsRBD with nanomolar affinity^25^. These two domains are connected to each other and to dsRBD3 by long and flexible linkers^30^. We speculate that this long, stretched structure makes TRBP a main entry port into Dicer for RNA. The high affinity of TRBP towards dsRNA molecules (*K*_D_=0.24 nM)^25^ together with its flexible and stretched structure allow to trap any type of dsRNA without selectivity, lowering the energy level of (i) and the energy barrier between (i) and (ii) in Fig. 7a. In return, Dicer moderates the binding affinity of TRBP for fast turnover. TRBP within the Dicer complex holds pre-miRNA momentarily (Fig. 5), more than two orders of magnitude shorter than when TRBP acts alone (Fig. 6), until it either transfers it to Dicer or releases it to the solution. We speculate that, by competing for RNA, RNA-interacting domains of Dicer might reduce the apparent RNA-binding affinity of TRBP. Alternatively, Dicer might cause steric hindrance to the RNA-binding domain of TRBP.

Why does RNA processing demand this intertwined coordination between Dicer and TRBP? Theoretical work has suggested that accurate target recognition can be time-efficient if two different modes of binding are coupled: a rapid mode in which a protein binds molecules promiscuously, which is followed by a subsequent slower but specific recognition mode^31^,^32^. Here we showed that the dual recognition mechanism of short binding (non-selective) and long binding (selective) provides both efficacy and fidelity in discriminating precursor miRNA from other cellular RNA species, including messenger RNA and non-coding RNA. When Dicer is decoupled from TRBP, Dicer is still capable of processing pre-miRNA. However, when it is challenged with a large amount of RNA structures that are abundant in the cell, Dicer lacking TRBP is recurrently stably associated with pre-miRNA-like substrates and cannot bind and process pre-miRNA efficiently anymore. We speculate that such a time-efficient recognition process might be used by other RNA-editing enzymes, including Dicer proteins from other species that partner with double-stranded RNA-binding cofactors and the nuclear microprocessor Drosha-DGCR8. It will be of great interest to verify our *in vitro* observation *in vivo*, for example, by extending previous TRBP depletion experiments^11^,^13^ to a time-controlled experiment, which will allow for tracing how the level of miRNA changes before a potential cellular compensation occurs^33^,^34^.

## Methods

### Cell culture and transfection

Human embryonic kidney cells (HEK 293, Sigma-Aldrich) were grown in Dulbecco's Modified Eagle's Medium (DMEM, Life Technology) supplemented with 10% fetal bovine serum (heat-inactivated, Greiner Bio-One) at 37 °C and 5% CO_2_. Before transfection, the cells were split into 10 cm cell culture dishes at 25% confluence. Then, 24 h after seeding, the plasmids were transfected using the CaPO_4_ method^35^. To allow for *in vivo* biotinylation of human Dicer, an additional plasmid coding for the BirA enzyme was co-transfected. After 5 h, the medium was exchanged with fresh DMEM containing 1 μg ml^−1^ biotin (Sigma-Aldrich), and the transfected cells were incubated for an additional 48 h to allow protein expression and *in vivo* biotinylation. Mycoplasma tests showed that our cells are not contaminated (MycoAlert Kit, Lonza).

### Cell harvest and lysis

Before collecting the cells, the DMEM was removed and the cells were washed with pre-chilled phosphate-buffered saline (PBS, pH 7.5, Life Technology). The cells were transferred to 15 ml tubes and centrifuged at 300*g* at 4 °C for 5 min to form cell pellets. After discarding the PBS supernatant, the cell pellets were kept frozen at −80 °C for long-term storage. For immediate lysis of freshly collected cells, the cell pellets were frozen for 30 min or longer at −80 °C for consistency.

Before cell lysis, the cells were thawed on ice for over 30 min and subsequently resuspended in buffer D (20 mM Tris (pH 8.0), 200 mM KCl and 0.2 mM EDTA). Lysis was carried out by carefully passing the cells 10 times through a needle (30½ gauge, BD) while avoiding formation of air bubbles using a 1 ml syringe. After cell lysis, the lysate was centrifuged twice (16,100*g* at 4 °C for 20 min) to remove cell debris (pellet). The recovered cell extract (supernatant) was used in tandem purification steps.

### Immunoprecipitation

For immunoprecipitation of FLAG-tagged proteins (wild type and TN Dicer), 1 mg of total protein in the cell extract was incubated 60 min with 2.5 μl of anti-FLAG antibody-conjugated agarose beads (50% slurry, anti-FLAG M2 affinity gel, Sigma-Aldrich) under gentle agitation at 4 °C. After the incubation, the beads were gently washed five times with buffer D and resuspended in 10 μl of buffer D. In cases when the lysis buffer was alternatively used, the buffer was replaced with buffer D at the last step. For the tandem purification, proteins were eluted out of the beads by site-specific cleavage with tobacco etch virus (TEV) protease (0.05 U μl^−1^; ProTEV Plus, Promega) at 30 °C for 90 min.

### Western blotting

Total cell extracts and immunopurified proteins were separated with 10% SDS–PAGE, transferred to polyvinylidene fluoride membranes (Millipore) and blocked with 5% skim milk (BD Difco). The membrane was exposed to primary antibodies in a 3% BSA solution at room temperature for 1 h or at 4 °C overnight. For chemiluminescence detection, horseradish peroxidase-conjugated secondary antibodies or streptavidin-horseradish peroxidase were incubated at room temperature for 30 min. Chemiluminescence was detected by incubating the membrane with the ECL solution (SuperSignal West Pico Chemiluminescence, PIERCE). The chemiluminescent blots were imaged with ChemiDoc MP imager (Bio-Rad). The antibodies and their dilution factors are listed in Supplementary Table 1.

### Immunofluorescence

Transfected cells were grown on poly-L-lysine-coated glass coverslips. Forty-eight hours after the transfection, cells were fixed with methanol during 10 min at −20 °C, and washed with pre-chilled PBS twice. Blocking and antibodies hybridization were performed in PBS containing 3% fetal calf serum and 0.1% Triton X-100. After 1 h of incubation with fluorophore-coupled antibodies (Supplementary Table 1) at room temperature, cells were washed three times with PBS, once with distilled water and finally mounted with Gel Mount solution. Immunofluorescence pictures were taken with a wide-field fluorescence microscope.

### Dicer processing

Dicer cleavage reactions were performed in a total volume of 20 μl in 10 mM Tris (pH 8.0), 0.1 mM EDTA, 100 mM KCl, 10 mM MgCl2, 1 mM dithiothreitol, 1 U ml^−1^ Ribonuclease inhibitor (Takara), 250–1,000 pM of internally Cy5-labelled pre-miRNA and 3 μl of the purified human Dicer. The reaction mixture was incubated at 37 °C for different incubation times. The RNA was purified from the reaction mixture by phenol extraction and separated on 10% urea polyacrylamide gel and was scanned with a Typhoon phosphorimager (GE Healthcare).

### RNA preparation and labelling

All pre-miRNA constructs used in this study were synthesized by ST-Pharm. The amine-modified pre-miRNAs were generated by ligation of two synthetic RNAs (Supplementary Table 2). First, an RNA single strand containing the 5p strand and a half of the terminal loop of the pre-miRNA (acceptor, 200 pmol) was mixed with the other strand containing the 3p strand and the other half of the terminal loop (donor, 100 pmol). The mixture (20 μl) in TE buffer with 100 mM NaCl was annealed by heating to 80 °C followed by slowly cooling (−1 °C per 4 min in a thermal cycler). The annealed substrate was ligated with 3 μl T4 RNA ligase (Ambion, 5 U μl^−1^), 3 μl 0.1% BSA, 5 μl 10 × ligation buffer provided and 19 μl H_2_O at 16 °C for 24 h. After ethanol precipitation, the RNA was purified with 12.5% urea polyacrylamide gel. The RNA strands were labelled with the NHS-ester form of Cy dyes (GE Healthcare) at an ∼100% efficiency^36^. We tested whether the position of the dye affected the cleavage by Dicer-TRBP. We generated four pre-let-7a-1^3′2nt^ constructs labelled at a different position and compared the cleavage efficiency (Supplementary Fig. 10). Unlike labelling at the single-stranded region of the loop (leading to an abnormal cleavage pattern and poor cleavage efficiency), labelling at the stem region was well tolerated by Dicer-TRBP (Supplementary Fig. 10). A construct with a dye at nt 69 (our standard construct) showed the highest cleavage efficiency.

### Microfluidic chamber

To eliminate non-specific surface adsorption of proteins and nucleic acids, the quartz surface (Finkenbeiner) of the microfluidic chamber was coated with poly-ethyleneglycol (mPEG-Succinimidyl Valerate, molecular weight 5,000, Laysan). A fraction of the poly-ethyleneglycol had biotin at the end (Biotin-PEG-SVA, molecular weight 5,000, Laysan). NeutrAvidin was layered on the surface via conjugation with the biotin. The details can be found elsewhere^36^. Finally, biotinylated Dicer IPs were specifically immobilized via the biotin–NeutrAvidin interaction. The binding between biotinylated Dicer and NeutrAvidin is stable for several hours without any noticeable dissociation.

### Single-molecule reaction

A volume of 50 μl of NeutrAvidin (100 μg ml^−1^, Invitrogen) was incubated for 1 min in the chamber. After washing unbound NeutrAvidin away with 100 μl buffer D, biotinylated Dicer IPs (20 μl) was incubated for 1 min in the chamber. After washing the unbound proteins away with 100 μl buffer D, 200 nM dye-labelled pre-miRNA was injected in the imaging buffer unless otherwise specified. The imaging buffer consisted of buffer D (20 mM Tris (pH 8.0), 200 mM KCl and 0.2 mM EDTA (pH 8.0)), an oxygen scavenging system (0.8% glucose (v/v), 0.1 mg ml^−1^ glucose oxidase (Sigma-Aldrich), 17 μg μl^−1^ catalase (Roche)) to reduce photobleaching and 1 mM Trolox (Sigma-Aldrich) to reduce photoblinking of the dyes^37^. To prevent direct binding of biotinylated pre-let-7a-1 (pre-let-7a-13′^2nt-biotin^) to the surface (Fig. 3), 50 μl of 1 mM biotin (dissolved in buffer D) was added to the imaging chamber to saturate all biotin-binding sites (Supplementary Fig. 7b).

To reach an accurate 1:1 ratio between the concentrations of two RNA samples in two-colour measurements, we measured sub-nanomolar concentrations of RNA using single-molecule fluorescence. We adsorbed RNA molecules to a positively charged surface as follows. KOH-etched quartz slides were coated with a layer of positively charged poly-L-lysine. After 5 min of incubation with 20 μl 0.01% poly-L-lysine (P4707, Sigma), the chamber was washed with 100 μl of buffer T50 (10 mM Tris (pH 8.0), 50 mM NaCl). After washing, two fluorescently labelled RNA substrates (modified: Cy3; reference: Cy5) were introduced into the microfluidic chamber. After 5 min of incubation, the unbound substrate was washed away with 100 μl of imaging buffer D and data were obtained from 10 fields of view. For each construct this procedure was repeated with three individual dilutions on three different slides.

### Single-molecule data acquisition

The fluorescent label Cy3 was imaged using prism-type total internal reflection microscopy at an excitation at 532 nm (Compass 215M-50, Coherent). Cy5 was excited by a 633 nm HeNe laser (CVI Melles Griot 25 LHP 928, 633 nm). When obtaining the time traces, we excited Cy3 and Cy5 molecules with 532 and 633 nm laser light sources, respectively, as weakly as possible to minimize Cy3 and Cy5 photobleaching during our observation. Under this imaging condition, only a minor fraction of the time traces were affected by Cy3 or Cy5 photobleaching or photoblinking during the first few minutes of imaging (Fig. 6e). Despite this precaution, the long-lived binding event might still be influenced by photobleaching and thus underrepresented in the population analysis.

Fluorescence signals from single molecules were collected with a × 60 water-immersion objective (UPlanSApo, Olympus) with an inverted microscope (IX71, Olympus). Scattering of the 532 nm laser beam was blocked with a 550 nm long-pass filter (LP03-532RU-25, SemRock). When the 633 nm laser was used, 633 nm laser scattering was blocked with a notch filter (NF03-633E-25, SemRock). Subsequently, the signals from Cy3 and Cy5 were spectrally split with a dichroic mirror (*λ*cutoff=645 nm, Chroma) and imaged onto two halves of an electron-multiplying charge-coupled device camera (iXon 897, Andor Technology). A series of charge-coupled device images were acquired with in-house software written in Visual C++ with a time resolution of 0.3 s. The CCD images illustrated the binding events over 25 × 25 μm^2^ field of view.

### Single-molecule data analysis

Fluorescence images and time traces were extracted with programmes written in IDL (ITT Visual Information Solutions) and analysed with Matlab (MathWorks) and Origin (OriginLab Corporation). To systematically select single-molecule fluorescence signals of Cy3 and Cy5 from the acquired images, we used an algorithm written in IDL that looked for fluorescence spots with a defined Gaussian profile and with signals above a threshold. This algorithm was effective in differentiating non-specific signals from other sources.

A dwell-time distribution was fitted by either a single-exponential decay curve 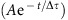 or a double-exponential decay curve 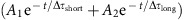. In case of a double-exponential decay, the percentages of 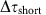 and 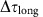 populations are determined by 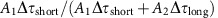 and 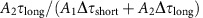, and the average dwell time is determined by 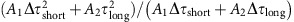.

### Data availability

The data and the computer codes that support the findings of this study are available from the authors on request.

## Additional information

**How to cite this article:** Fareh, M. *et al*. TRBP ensures efficient Dicer processing of precursor microRNA in RNA-crowded environments. *Nat. Commun.* **7,** 13694 doi: 10.1038/ncomms13694 (2016).

**Publisher's note:** Springer Nature remains neutral with regard to jurisdictional claims in published maps and institutional affiliations.

## Supplementary Material

Supplementary Information Supplementary Figures 1-10 and Supplementary Tables 1-2

Supplementary Movie 1 The movie displays a two-color competition assay (obtained with a time resolution 300 msec). The standard pre-let-7a-13' 2nt was labeled with Cy5 (right). A biotin group was attached to the 3' overhang of Cy3-labeled pre-let-7a-1 (pre-let-7a-13' 2nt-biotin; left). The movie was obtained by flow measurement in a pre-steady-state (0 min) and in a steady-state condition (80 min). 200 pM of both RNA substrates was used.

Peer Review File
