# Supplementary material for: TRBP ensures efficient Dicer processing of precursor microRNA in RNA-crowded environments
Source: Nat Commun. 2016 Dec 9;7:13694. doi: 10.1038/ncomms13694 (PMC5155159; doi:10.1038/ncomms13694)
Supplement: Supplementary Information — Supplementary Figures 1-10 and Supplementary Tables 1-2 [file ncomms13694-s1.pdf]

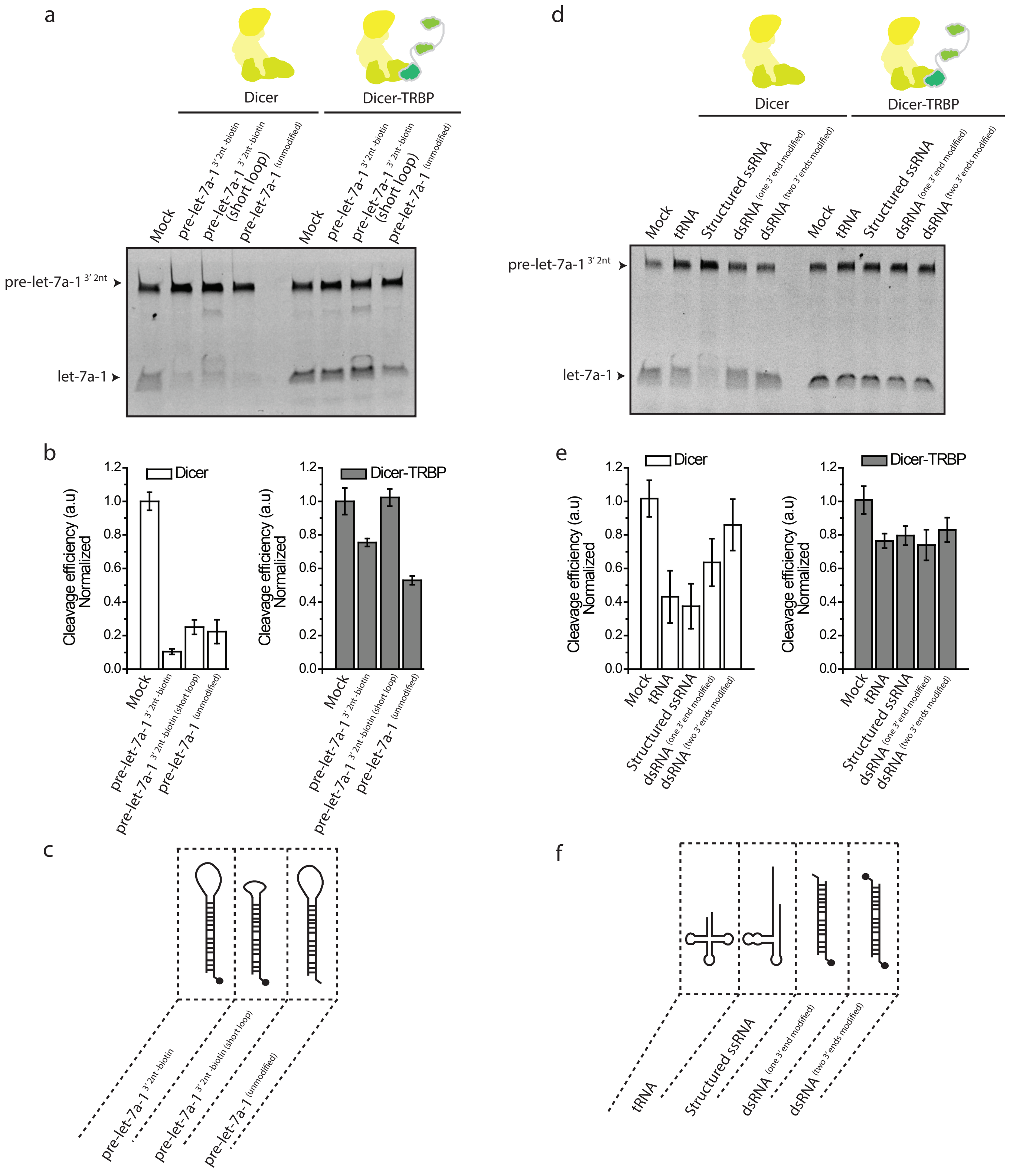

Supplementary Figure 1. TRBP ensures efficient processing of pre-miRNAs in an RNA crowded cellular environment

(a) In vitro cleavage of pre-let-7a-1$^{3'\,2nt}$ (1 nM) by Dicer alone or Dicer-TRBP complex. The cleavage was performed with an excess of pre-miRNA-like substrates (500 nM).

(b) Quantification of the cleavage efficiency. A quencher, pre-let-7a-1$^{3'\,2nt}$ with biotinylated 3' end, led to substantial inhibition (by 90%) of Dicer-alone cleavage activity but only moderate inhibition (by 20%) of Dicer-TRBP activity. pre-let-7a-1$^{3'\,2nt}$ with biotinylated 3' end and a short loop also led to substantial inhibition (by 80%) of Dicer-alone cleavage activity but no inhibition of Dicer-TRBP activity. The canonical pre-let-7a-1 (72nt) inhibited the cleavage activity of both Dicer-alone (by 80%) and Dicer-TRBP (by 50%).

(c) The structure of the quenchers. pre-let-7a-1$^{3'\,2nt\text{-}biotin}$ contains a non-canonical 3' end (biotin). pre-let-7a-1$^{3'\,2nt\text{-}biotin\ (short\ loop)}$ contains a non-canonical 3' end (biotin) and a short terminal loop (4 nt). The third one is a canonical pre-miRNA (pre-let-7a-1, 72nt).

(d) In vitro cleavage of pre-let-7a-1$^{3'\,2nt}$ (1 nM) by Dicer-alone or Dicer-TRBP complex. The cleavage was performed with an excess of dsRNA substrates (500 nM).

(e) Quantification of the cleavage efficiency. tRNA and a structured ssRNA showed substantial inhibition (by 60%) of Dicer-alone cleavage activity but only moderate inhibition (by 20%) of Dicer-TRBP activity. Duplex RNA of 22nt with one biotinylated 3' end exhibited a partial inhibition of Dicer cleavage activity (by 40%) whereas duplex RNA with two biotinylated 3' ends only moderately suppressed the cleavage activity (by 20%) indicating the importance of the 3' end of RNA for Dicer-RNA interaction.

(f) Schematic representation of the structure of RNA quenchers.

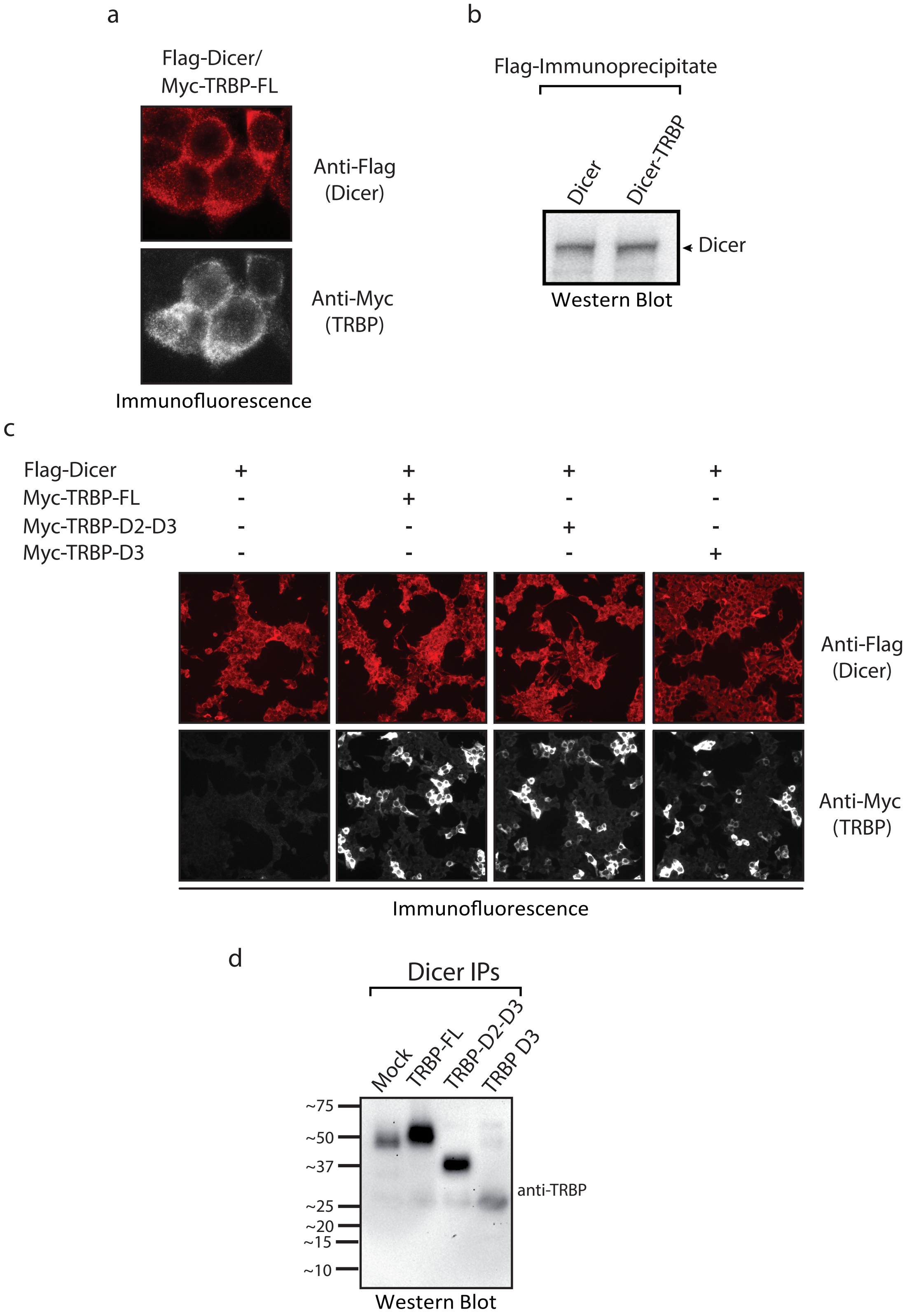

Supplementary Figure 2. TRBP proteins

(a) Immunofluorescence analysis shows the expression and cellular colocalization of Flag-Dicer (upper panel) and Myc-TRBP (lower panel) within the P-bodies of HEK 293 cells.

(b) Western blotting of Dicer immunoprecipitates (IPs) to quantify Dicer enrichment. Dicer proteins were expressed without and with full length TRBP and were pulled down using Flag-beads.

(c) Immunofluorescence analysis shows the expression level of Flag-Dicer (upper panels) and different forms of Myc-TRBP (lower panels) in 293T transfected cells.

(d) Western blot analysis of TRBP truncated forms associated with Dicer. A very small amount of the endogenous TRBP was co-immunoprecipitated with ectopically expressed Dicer (lane 1). TRBP full length and TRBP D2-D3 (lanes 2 and 3 respectively) were associated to Dicer. TRBP-D3 was detected at a lower level (lane 4).

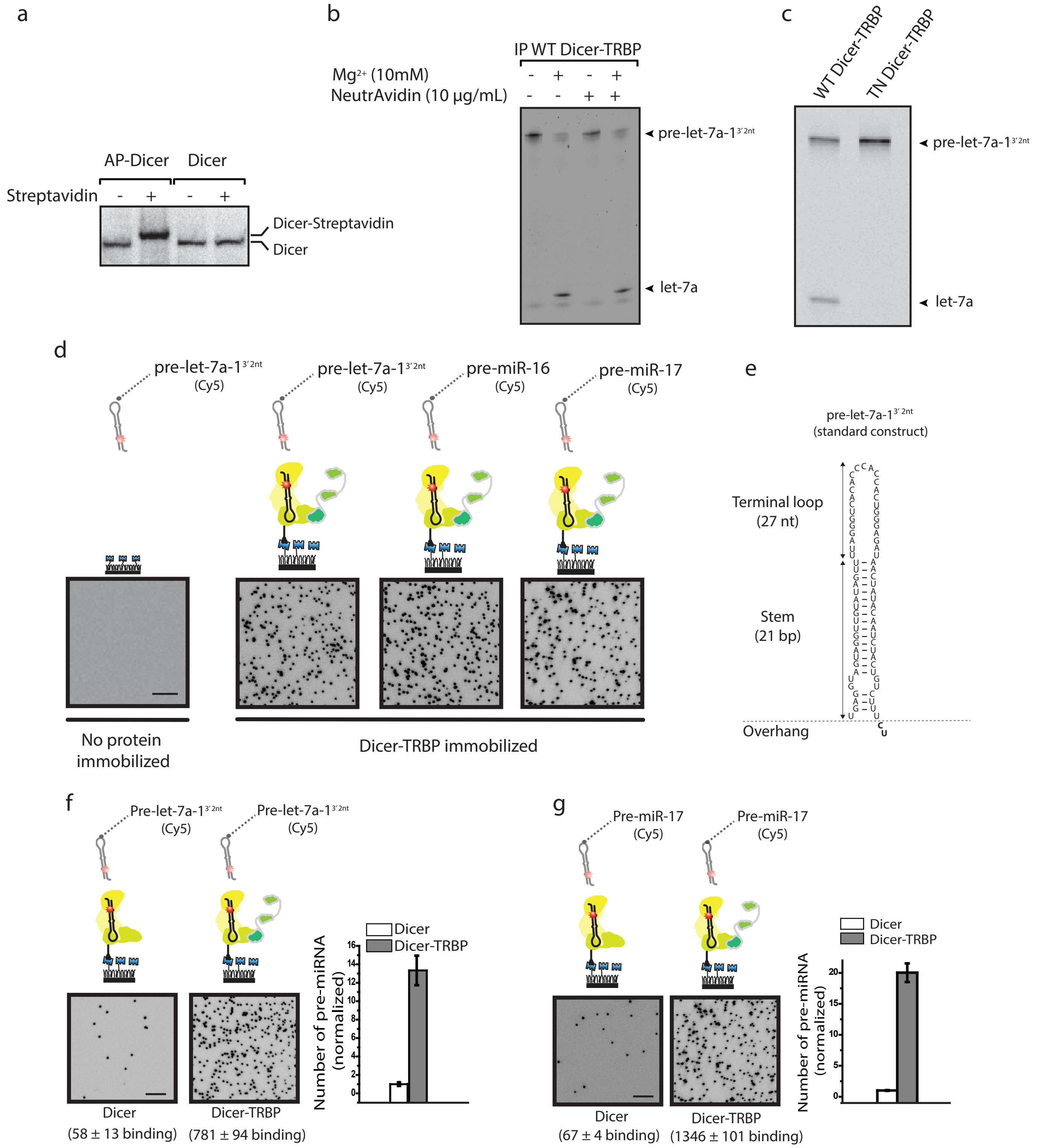

Supplementary Figure 3. Single-molecule immunoprecipitation reveals the role of TRBP in pre-miRNA recruitment by Dicer-TRBP complex

(a) The western blot analysis displays the efficiency of *in vivo* biotinylation of AP-tagged (lanes 1 and 2) and untagged (lanes 3 and 4) Dicer. The biotinylated Dicer bound to streptavidin, which resulted in the shift observed in lane 2.

(b) In vitro cleavage assay of standard pre-let-7a-1$^{3'\,2nt}$ by WT Dicer-TRBP in absence (lanes 1 and 2) and presence of NeutrAvidin (lanes 3 and 4). The top arrow indicates pre-let-7a-1$^{3'\,2nt}$, and the bottom arrow indicates a cleaved product (let-7a).

(c) In vitro cleavage assay of standard pre-let-7a-1$^{3'\,2nt}$ by WT Dicer-TRBP or TN Dicer-TRBP (catalytically inactive protein). pre-let-7a-1$^{3'\,2nt}$ was incubated 30 min at 37°C with WT Dicer-TRBP or TN Dicer-TRBP immunoprecipitates (IPs). The top arrow indicates pre-let-7a-1$^{3'\,2nt}$, and the bottom arrow indicates a cleaved product (mature let-7a).

(d) CCD images display the RNA binding activity of Dicer-TRBP complex. As a negative control, Cy5-labeled pre-let-7a-1$^{3'\,2nt}$ was introduced into a passivated surface without Dicer immobilized (left panel). Surface-immobilized Dicer-TRBP complex stably bound to pre-let-7a-1, pre-miR-16 and pre-miR-17 (right panels). Scale bar, 5 μm. 200 pM of RNA substrates was used.

(e) Structure of standard pre-let-7a-1 with 3' 2nt overhang.

(f) Quantification of the number of pre-let-7a-1$^{3'\,2nt}$ stably bound to TN Dicer-alone (CCD image, left panel) or TN Dicer-TRBP (CCD image, right panel) in the steady-state condition. Scale bar, 5 μm. The histogram displays a normalized number of binding events obtained with an equal amount of IPs of TN Dicer (white) or TN Dicer-TRBP (gray). 200 pM of pre-let-7a-1$^{3'\,2nt}$ was used.

(g) Quantification of the number of pre-miR-17 stably bound to TN Dicer-alone (CCD image, left panel) or TN Dicer-TRBP (CCD image, right panel) in the steady-state condition. Scale bar, 5 μm. The histogram displays a normalized number of binding events obtained with an equal amount of IPs of TN Dicer-alone (white) or TN Dicer-TRBP (gray). 200 pM of pre-miR-17 was used.

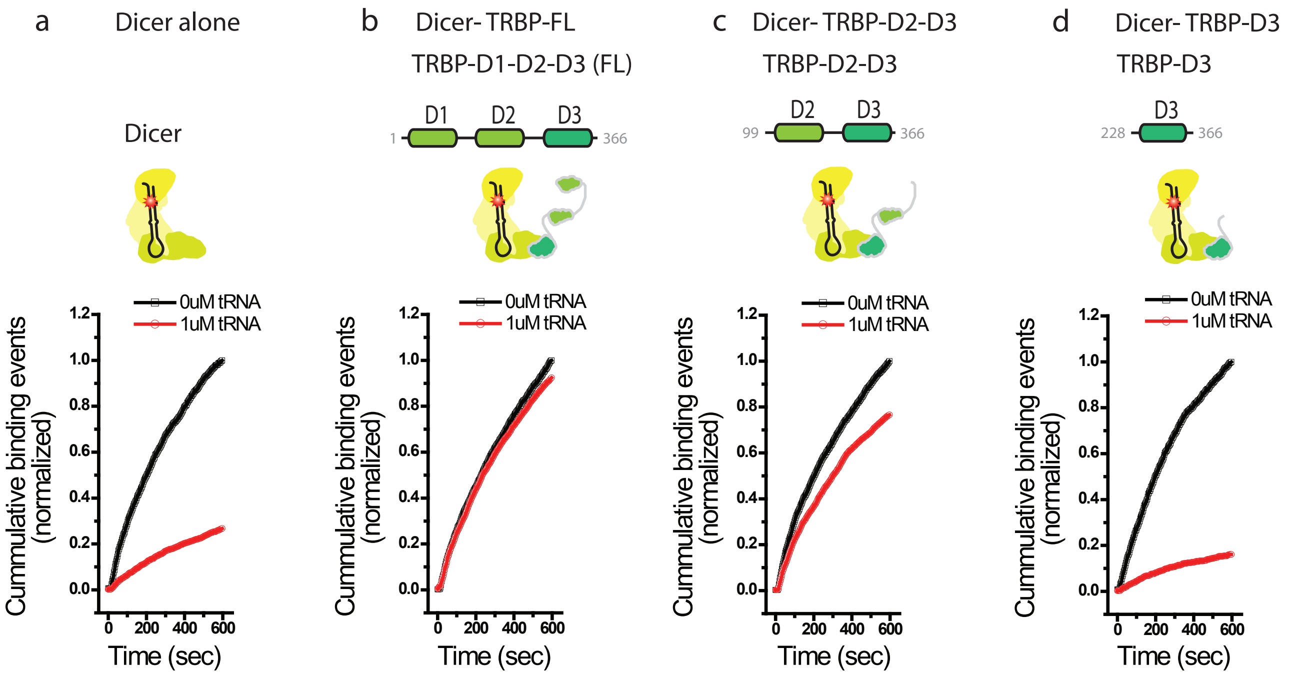

Supplementary Figure 4. TRBP's dsRBDs mediate the recruitment of pre-miRNA in an RNA crowded environment

(a-d) Single-molecule assay to capture the recognition of pre-let-7a-1$^{3'\ 2nt}$ (200 pM) by Dicer alone (a), Dicer-TRBP-FL (b), Dicer-TRBP-D2-D3 (c) and Dicer-TRBP-D3 (d) in absence (black) and presence of 1 µM competitor tRNA (red). TRBP constructs used are shown in the top panels. The first and last amino acid numbers of each construct are specified. FL indicates full length. The bottom graphs show normalized cumulative plots of the binding events between Dicer complexes and pre-let-7a-1$^{3'\ 2nt}$ during the first 600 seconds.

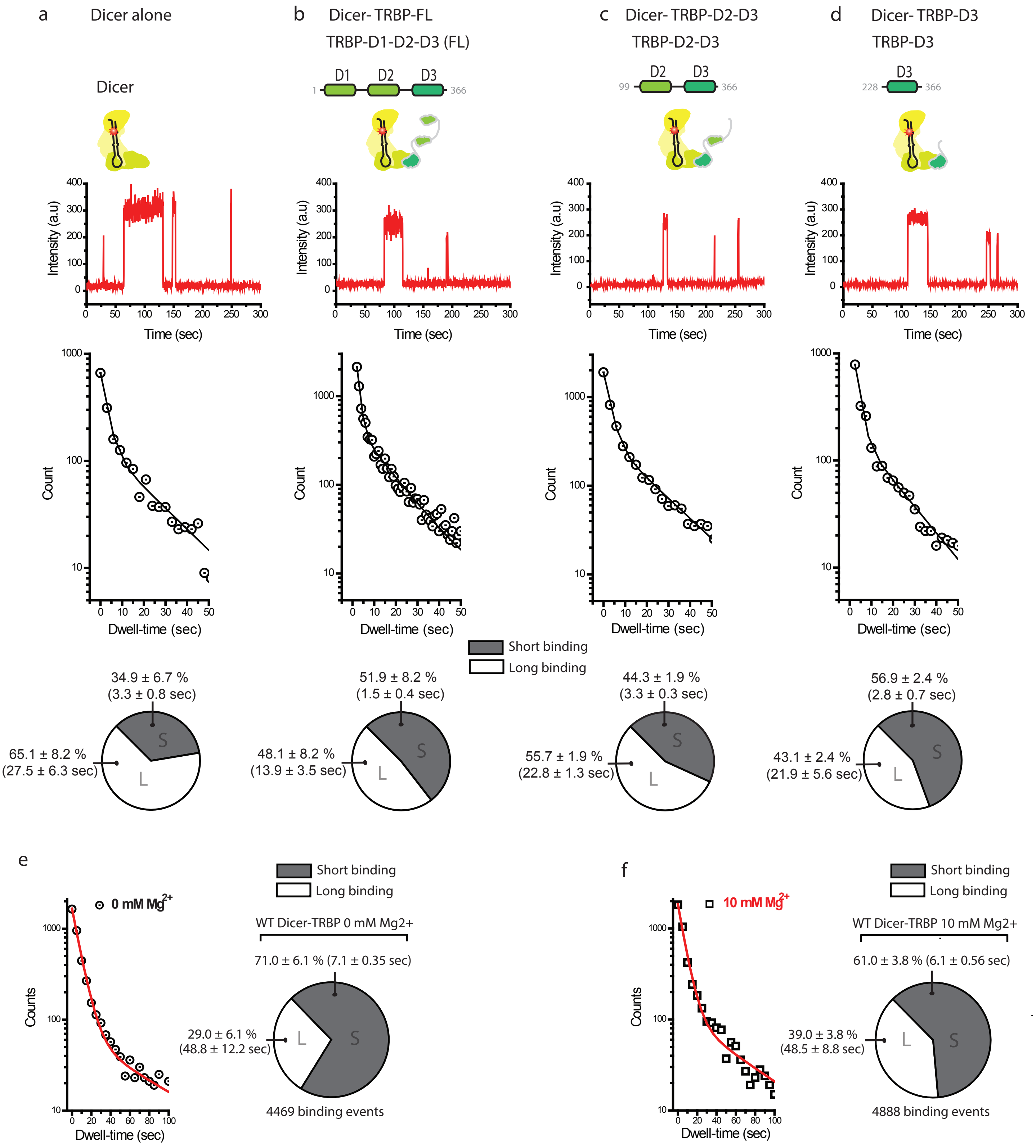

Supplementary Figure 5. Binding modes of Dicer alone or Dicer associated with different forms of TRBP

(a-d) Representative time traces and dwell-time histograms derived from binding of standard pre-let-7a-1$^{3'\ 2nt}$ to surface immobilized (a) Dicer alone, (b) Dicer-TRBP-FL, (c) Dicer-TRBP-D2-D3 and (d) Dicer-TRBP-D3. The distributions were fitted with a double exponential decay. The pie charts in the bottom display the percentage of short binding (gray) and long binding (white) obtained with Cy5-labeled standard pre-let-7a-1$^{3'\ 2nt}$. Error is the SD of three independent measurements. To obtain a sufficient number of binding events when assessing the effect of TRBP on the binding modes, we used a four-fold larger amount of TN Dicer alone and TN Dicer-TRBP-D3 IPs, and a two-fold larger amount of TN Dicer-TRBP-D2-D3 IP, compared to that of Dicer-TRBP-FL IP.

(e-f) Dwell-time histograms obtained from WT-Dicer-TRBP interaction with 200 pM standard Cy5-labeled pre-let-7a-1$^{3'\ 2nt}$ in absence and presence of 10 mM MgCl2. The pie chart in the left displays the percentage of short binding ($\Delta\tau$short = 7.1 ± 0.35 sec, gray) and long binding ($\Delta\tau$long = 48.8 ± 12.2 sec, white) obtained without MgCl2. The pie chart in the right displays the percentage of short binding ($\Delta\tau$short = 6.1 ± 0.56 sec, gray) and long binding ($\Delta\tau$long = 48.5 ± 8.8 sec, white) obtained with 10 mM MgCl2.

To summarize these data, we note that our standard system is a catalytically dead mutant TN-Dicer. We observed that the wild type (WT) Dicer-TRBP also exhibited the two binding modes (Supplementary Fig. 5e-f). We additionally tested whether magnesium ions influence the substrate recognition. Ribonucleases including Dicer require magnesium ions to cleave RNA. When the magnesium was depleted, two binding modes persisted, and the dwell-times of the two binding modes remained unchanged. The total number of binding events was also similar (Supplementary Fig. 5e-f).

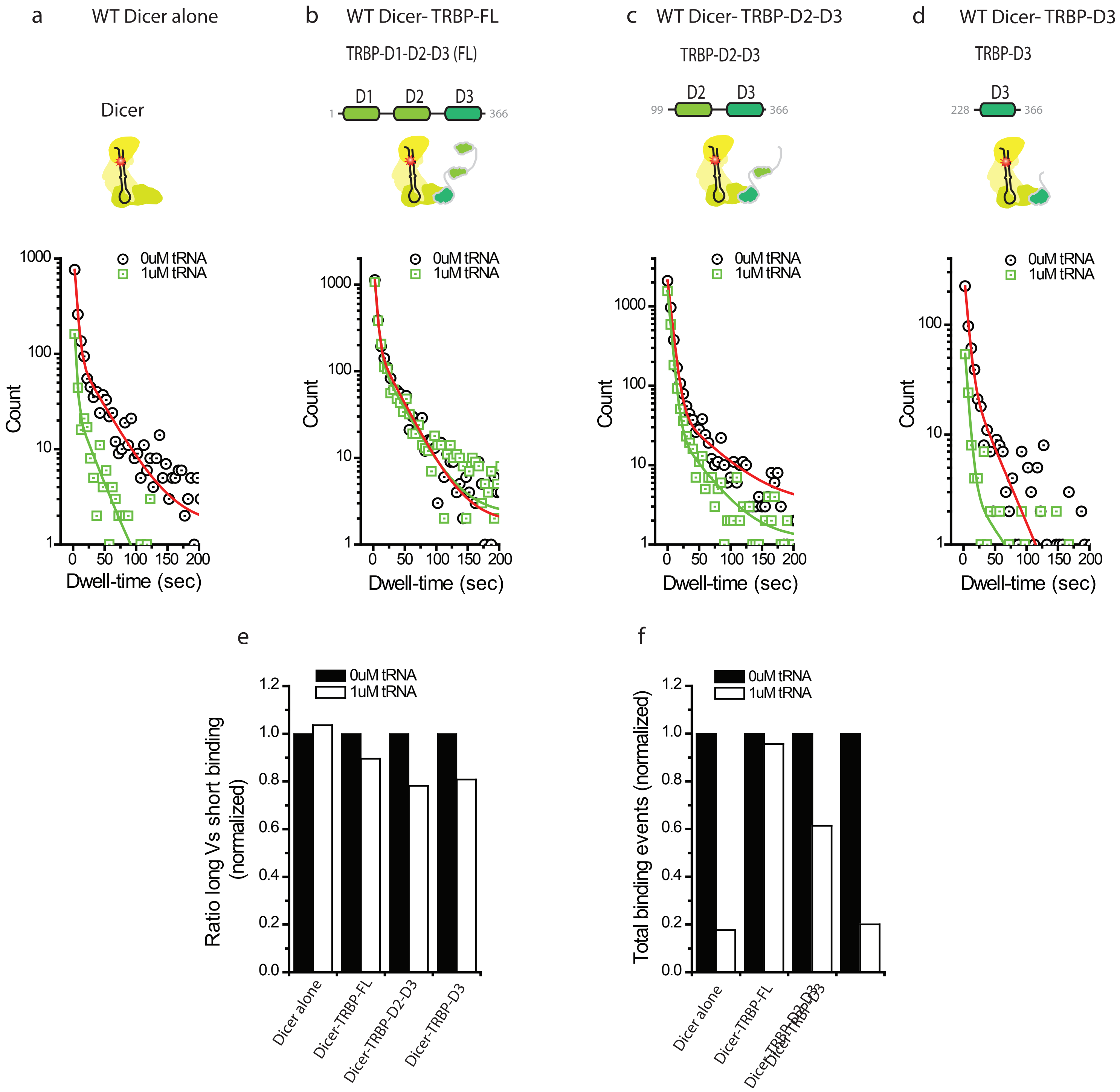

Supplementary Figure 6. dsRBD of TRBP mediate the recruitment of pre-miRNA in RNA crowded environment

(a-d) Dwell-time histograms of pre-let-7a-1$^{3' 2nt}$ (200 pM) interaction with (a) WT-Dicer alone, (b) WT-Dicer-TRBP-FL, (c) WT-Dicer-TRBP-D2-D3 and (d) WT-Dicer-TRBP-D3 in presence (green) and absence (black) of excess competitor tRNA (1 μM). (e) The graph displays a normalized ratio between long and short binding in absence (black) and presence (white) of excess competitor tRNA. (f) The graph displays a normalized quantification of the overall binding events in absence (black) and presence (white) of excess competitor tRNA.

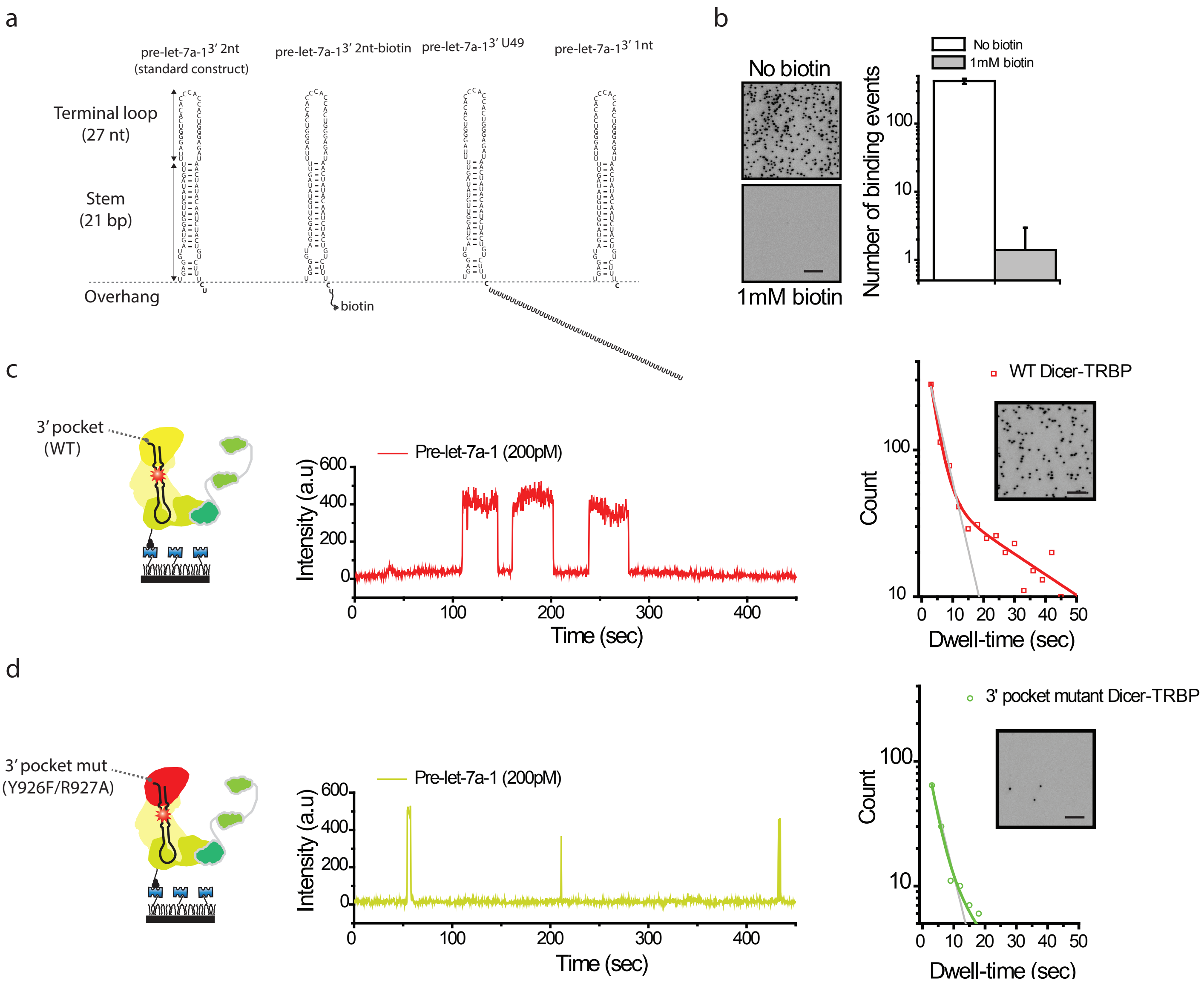

Supplementary Figure 7. The effect of PAZ domain mutation

(a) Structure of pre-let-7a-1 constructs used to investigate the recognition of 3' end by the PAZ domain.

(b) Direct surface immobilization of 100 pM of 3'end biotinylated pre-let-7a-1$^{3'\ 2nt}$ substrates (pre-let-7a-1$^{3'\ 2nt\text{-}biotin}$) before and after surface saturation with 1 mM free biotin. Scale bar, 5 µm.

(c) Representative time trace (time resolution 300 ms) showing binding of three standard pre-let-7a-1$^{3'\ 2nt}$ substrates (red) to a single wild type Dicer-TRBP complex. Dwell-time histogram derived from binding of 200 pM of standard pre-let-7a-1$^{3'\ 2nt}$. The distribution was fitted with a double exponential decay (red fit) and shows both short and long binding events. The CCD image shows stable docking of standard pre-let-7a-1$^{3'\ 2nt}$ to wild type Dicer-TRBP (left). Scale bar, 5 µm.

(d) Representative time trace (time resolution 300 msec) showing binding of three standard pre-let-7a-1$^{3'\ 2nt}$ substrates (green) to a single 3' pocket PAZ domain mutant Dicer-TRBP complex. Dwell-time histogram derived from binding of 200 pM of standard pre-let-7a-1$^{3'\ 2nt}$ (green). The distribution was fitted with a double exponential decay (green fit) and shows only short binding events. The CCD image shows the stable docking of standard pre-let-7a-1$^{3'\ 2nt}$ to 3' pocket PAZ domain mutant Dicer-TRBP complex (right). Scale bar, 5 µm.

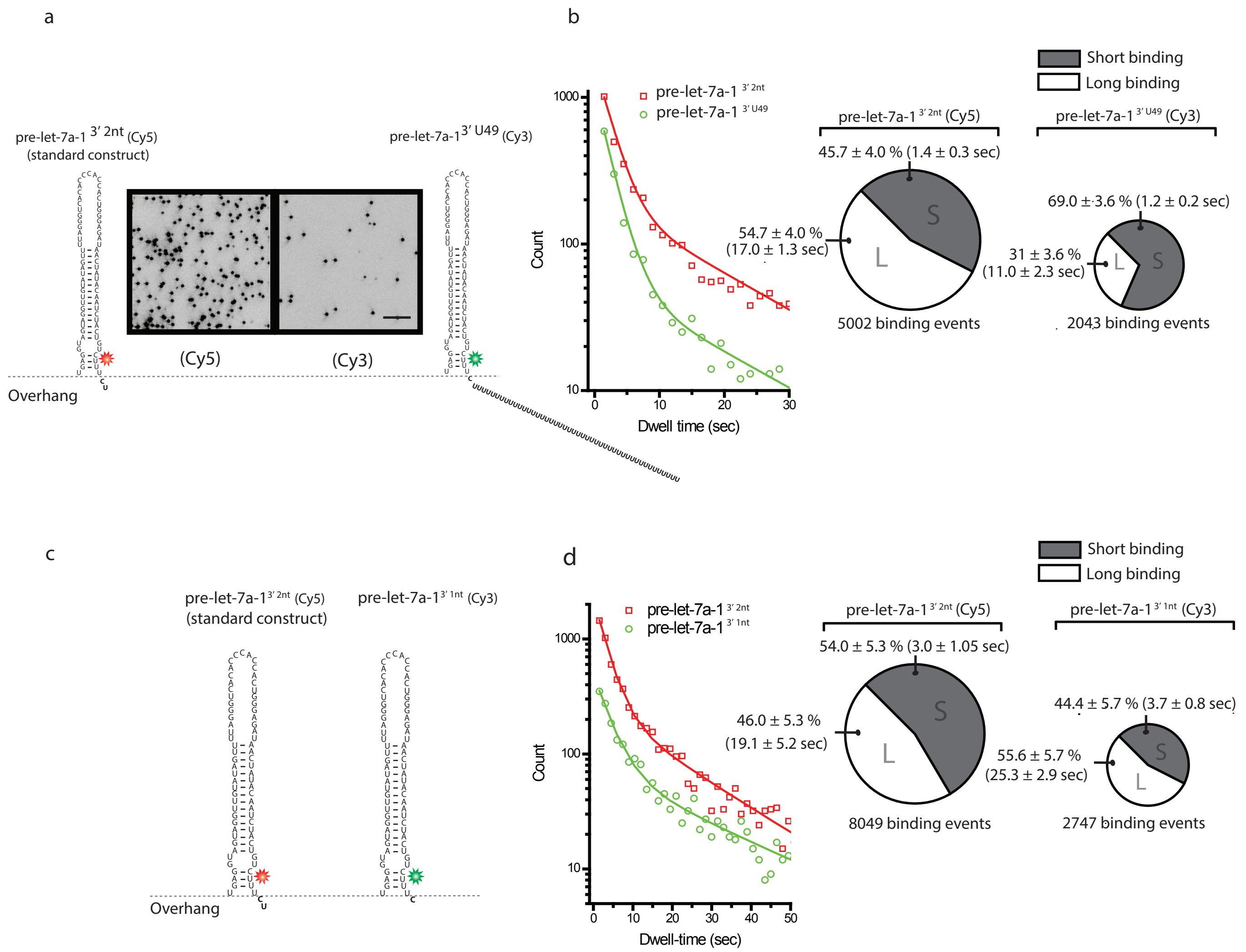

Supplementary Figure 8. The role of the PAZ domain

(a) Two-color competition assay. A standard Cy5-labeled pre-let-7a-1$^{3'\ 2nt}$ was used as control (left). U49 tail was attached to the 3' end of Cy3-labeled pre-let-7a-1 (pre-let-7a-1$^{3'\ U49}$) (right). The CCD images show stable docking of standard pre-let-7a-1$^{3'\ 2nt}$ (left) and pre-let-7a-1$^{3'\ U49}$ (right) to Dicer-TR-BP in a steady-state condition . Scale bar, 5 μm. 200 pM of RNA substrates was used.

(b) Dwell-time histograms derived from standard pre-let-7a-1$^{3'\ 2nt}$ (red) and pre-let-7a-1$^{3'\ U49}$ (green). The pie chart in the left displays the percentage of short binding (Δτshort = 1.4 ± 0.3 sec, gray) and long binding (Δτlong = 17.0 ± 1.3 sec, white) obtained with Cy5-labeled standard pre-let-7a-1$^{3'\ 2nt}$. The pie chart in the right is for short binding (Δτshort = 1.2 ± 0.2 sec, gray) and long binding (Δτlong = 11.0 ± 2.3 sec, white) obtained with Cy3-labeled pre-let-7a-1$^{3'\ U49}$. Error is the SD of four independent measurements. 200 pM of RNA substrates was used.

(c) Two-color competition assay. A standard Cy5-labeled pre-let-7a-1$^{3'\ 2nt}$ was used as control (left). pre-let-7a-1 with one nucleotide overhang (pre-let-7a-1$^{3'\ 1nt}$) was labeled with Cy3 (right). 200 pM of RNA substrates was used.

(d) Dwell-time histograms derived from standard pre-let-7a-1$^{3'\ 2nt}$ (red) and pre-let-7a-1$^{3'\ 1nt}$ (green). The pie chart in the left displays the percentage of short binding (Δτshort = 3.0 ± 1.05 sec, gray) and long binding (Δτlong = 19.1 ± 1.3 sec, white) obtained with Cy5-labeled standard pre-let-7a-1$^{3'\ 2nt}$. The pie chart in the right is for short binding (Δτshort = 3.7 ± 0.8 sec, gray) and long binding (Δτlong = 25.3 ± 2.9 sec, white) obtained with Cy3-labeled pre-let-7a-1$^{3'\ 1nt}$. Error is the SD of four independent measurements. 200 pM of RNA substrates was used.

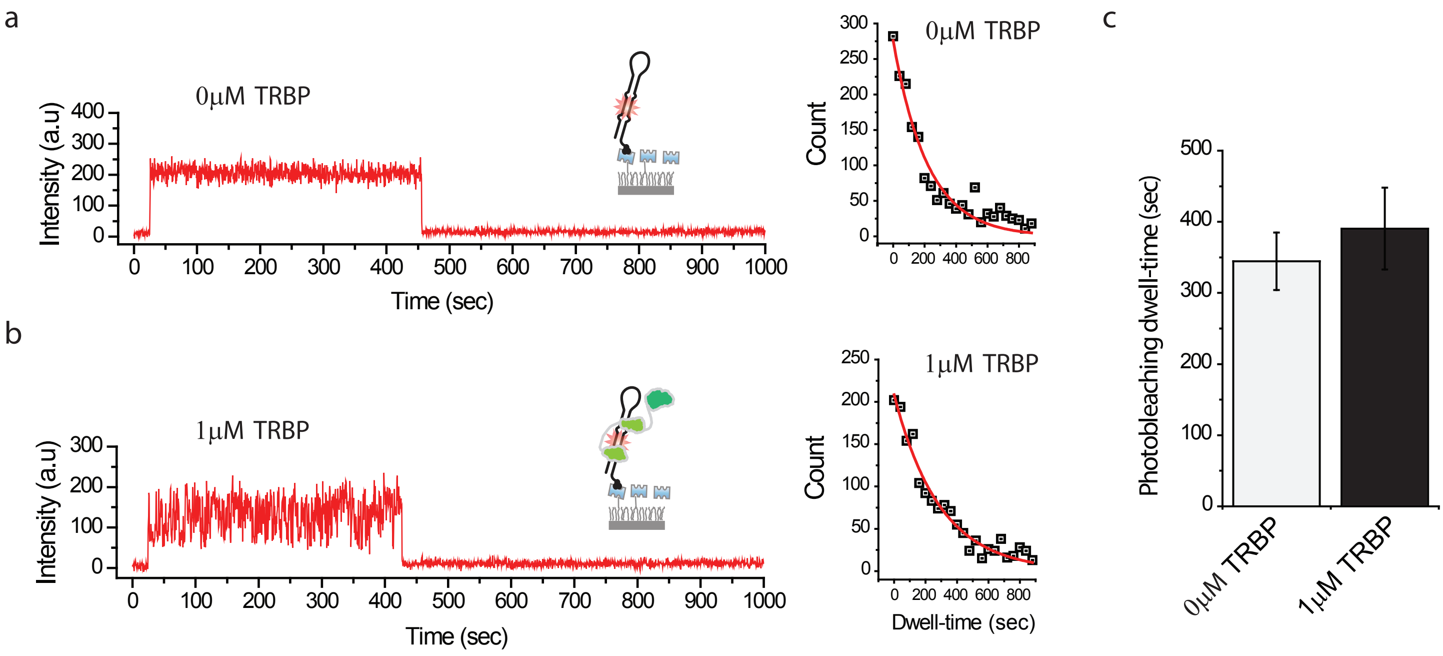

Supplementary Figure 9. TRBP's effect on the photobleaching of the Cy5 fluorophore

(a) Representative time trace obtained from direct immobilization of Cy5-labeled pre-let-7a-1$^{3' \text{ 2nt-biotin}}$ via biotin-NeutrAvidin conjugation. The loss of fluorescence reflects the photobleaching of the Cy5. The dwell-time histogram (right) derived from the photobleaching of surface-immobilized pre-let-7a-1$^{3' \text{ 2nt-biotin}}$. (b) Representative time trace obtained from direct immobilization of Cy5-labeled pre-let-7a-1$^{3' \text{ 2nt-biotin}}$ in presence of saturating concentration of recombinant TRBP (1 μM). The dwell-time histogram derived from the photobleaching of surface-immobilized pre-let-7a-1$^{3' \text{ 2nt-biotin}}$ in presence of recombinant TRBP (1 μM). (c) Average photobleaching dwell-times from [a] and [b]. Error is the SD of three experiments.

a

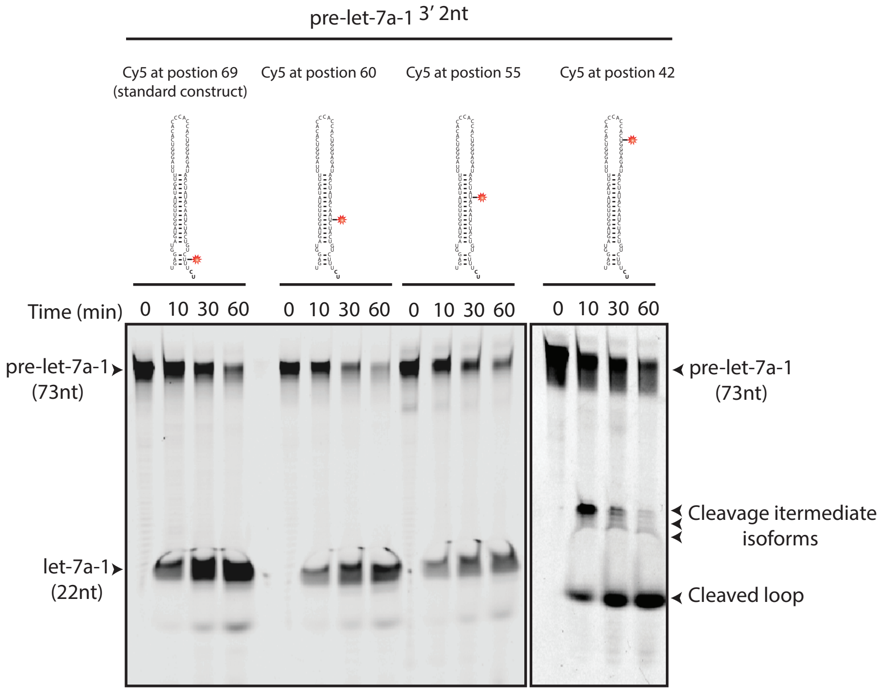

b

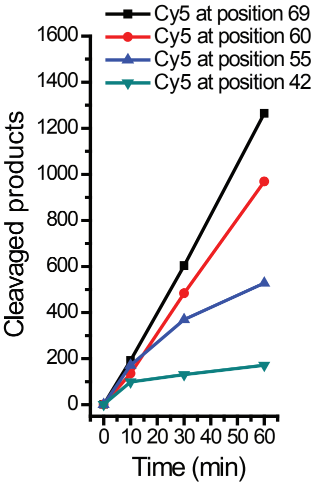

Supplementary Figure 10. Effect of the fluorophore position to the cleavage efficiency of Dicer

(a) Time course analysis of *in vitro* cleavage of four different pre-let-7a-1$^{3' 2nt}$ constructs labeled with Cy5 at different positions (69, 60, 55 and 42 position). (b) Quantification of the cleavage efficiency of the pre-let-7a-1$^{3' 2nt}$ constructs by WT Dicer-TRBP.

Supplementary Table 1. Antibodies used.

Antibodies and Streptavidin-HRP	Dilution factor
Rabbit anti-Dicer (polyclonal, Rnomics)	1/1000
Rabbit anti-cMyc (polyclonal, sc-789, Santa Cruz)	1/1000
Streptavidin-HRP (S2438-250UG Sigma-Aldrich)	1/1000
Goat anti-rabbit IgG-HRP (111-035-144, Jackson ImmunoResearch Laboratories)	1/10000
Donkey anti-mouse IgG-HRP, (715-035-150, Jackson ImmunoResearch Laboratories)	1/10000
Mouse Anti- DDDDK (Flag) DyLight® 550 (Monoclonal, ab117495, Abcam)	1/500
Mouse Anti-c-Myc DyLight® 650 (Monoclonal, ab117487, Abcam)	1/500

Supplementary Table 2. RNA sequences used.

Name	Sequence
pre-let-7a-1 (5p)	5'-UGA GGU AGU AGG UUG UAU AGU UUU AGG GUC ACA CC-3'
pre-let-7a-1$^{\text{3' 1nt}}$ (3p) (dye at position 69)	5'-pCAC CAC UGG GAG AUA ACU AUA CAA UCU ACU GUC ***u***UU C-3'
**pre-let-7a-1$^{\text{3' 2nt}}$ (3p) (standard construct, dye at position 69)**	5'-pCAC CAC UGG GAG AUA ACU AUA CAA UCU ACU GUC ***u***UU CU-3'
pre-let-7a-1$^{\text{3' 2nt}}$ (3p) (dye at position 60)	5'-pCAC CAC UGG GAG AUA ACU AUA CAA ***u***CU ACU GUC UUU CU-3'
pre-let-7a-1$^{\text{3' 2nt}}$ (3p) (dye at position 55)	5'-pCAC CAC UGG GAG AUA ACU A***u***A CAA UCU ACU GUC UUU CU-3'
pre-let-7a-1$^{\text{3' 2nt}}$ (3p) (dye at position 42)	5'-pCAC CAC ***u***GG GAG AUA ACU AUA CAA UCU ACU GUC UUU CU-3'
pre-let-7a-1$^{\text{3' 2nt-biotin}}$ (3p) (dye at position 69)	5'-pCAC CAC UGG GAG AUA ACU AUA CAA UCU ACU GUC ***u***UU CU-biotin-3'
pre-let-7a-1$^{\text{3' U49}}$ (3p) (dye at position 69)	5'-pCAC CAC UGG GAG AUA ACU AUA CAA UCU ACU GUC ***u***UU CUU UUU UUU UUU UUU UUU UUU UUU UUU UUU UUU UUU UUU UUU UUU UUU UU-3'
pre-miR-16-1 (5P)	5'-UAG CAG CAC GUA AAU AUU GGC GUU AAG AUU CUA-3'
pre-miR-16-1 (3P)	5'-pAAA UUA UCU CCA G***u***A UUA ACU GUG CUG CUG A-3'
pre-miR-17 (5P)	5'-CAA AGU GCU UAC AGU GCA GGU AGU GAU AUG-3'
pre-miR-17 (3P)	5'-pUGC AUC UAC ***u***GC AGU GAA GGC ACU UGU AG-3'
pre-let-7a-1$^{\text{3' 2nt-biotin short loop}}$ (5P)	5'-UGA GG***u*** AGU AGG UUG UAU AGU UUU-3'
pre-let-7a-1$^{\text{3' 2nt-biotin short loop}}$ (3P)	5'-pAUA ACU AUA CAA UCU ACU GUC UUU CU-biotin-3'
dsRNA $^{\text{(one 3'end modified)}}$	Sense 5'-UGA GG***u*** AGU AGG UUG UAU AGU U-biotin-3'
	Anti-sense 5'-CUA UAC AAU CUA CUG UCU UUC U-3'
dsRNA $^{\text{(two 3'end modified)}}$	Sense 5'-UGA GG***u*** AGU AGG UUG UAU AGU U-biotin-3'
	Anti-Sense 5'-CUA UAC AAU CUA CUG UCU UUC U-biotin-3'
Structured ssRNA	5'-GGA CCU CGG CGA AAG CUA UUC GAA ACG CGA AAG CAC UUA GAU GUG AGG UUA GGU GC-3’

u indicates dye-labeled position.
