# Supplementary material for: TRBP ensures efficient Dicer processing of precursor microRNA in RNA-crowded environments
Source: Nat Commun. 2016 Dec 9;7:13694. doi: 10.1038/ncomms13694 (PMC5155159; doi:10.1038/ncomms13694)
Supplement: Peer Review File [file ncomms13694-s3.pdf]

Editorial Note: this manuscript has been previously reviewed at another journal that is not operating a transparent peer review scheme. This document only contains reviewer comments and rebuttal letters for versions considered at Nature Communications.

Reviewers' Comments:

Reviewer #1 (Remarks to the Author)

The authors have adequately addressed my previous concerns and the manuscript is now ready for publication at Nature Communications.

Reviewer #2 (Remarks to the Author)

I will not describe the main achievements of the paper, since this was done in the review of the original submission. On the whole it has been significantly improved and the authors have experimentally addressed most of the reviewers concerns. There are no further concerns about the content and presentation of the manuscript in its current form. The result is a clear and strong manuscript!

Reviewer #3 (Remarks to the Author)

The authors have made an excellent effort to address all the questions and removing the cleavage analysis has made the work more clear and self-contained. Therefore at this stage I recommend its publication with no further comments
